# Future perspectives in melanoma research “Melanoma Bridge”, Napoli, November 30th–3rd December 2016

**DOI:** 10.1186/s12967-017-1341-2

**Published:** 2017-11-16

**Authors:** Paolo A. Ascierto, Sanjiv S. Agarwala, Gennaro Ciliberto, Sandra Demaria, Reinhard Dummer, Connie P. M. Duong, Soldano Ferrone, Silvia C. Formenti, Claus Garbe, Ruth Halaban, Samir Khleif, Jason J. Luke, Lluis M. Mir, Willem W. Overwijk, Michael Postow, Igor Puzanov, Paul Sondel, Janis M. Taube, Per Thor Straten, David F. Stroncek, Jennifer A. Wargo, Hassane Zarour, Magdalena Thurin

**Affiliations:** 1 Unit of Melanoma, Cancer Immunotherapy and Innovative Therapy, IRCCS Istituto Nazionale Tumori “Fondazione G. Pascale”, Naples, Italy; 2 Oncology & Hematology, St. Luke’s University Hospital and Temple University, Bethlehem, PA USA; 3 IRCCS “Regina Elena” National Cancer Institute, Rome, Italy; 4 Radiation Oncology and Pathology, Weill Cornell Medical College, New York City, NY USA; 5 Department of Dermatology, University of Zurich Hospital, Zurich, Switzerland; 6 INSERM (National Institute of Health and Medical Research), Institut Gustave Roussy, Villejuif, France; 7 Massachusetts General Hospital, Boston, MA USA; 8 Department of Radiation Oncology, Weill Cornell Medical College, New York City, NY USA; 9 Division of Dermatologic Oncology, Department of Dermatology, Eberhard Karls University, Tübingen, Germany; 10 Department of Dermatology, Yale University School of Medicine, New Haven, CT USA; 11 Georgia Cancer Center, Augusta University, Augusta, GA USA; 12 Department of Hematology/Oncology, University of Chicago Comprehensive Cancer Center, Chicago, IL USA; 13 CNRS (National Center for Scientific Research, France), University Paris-Saclay, Gustave Roussy, Villejuif, France; 14 Division of Cancer Medicine, Department of Melanoma Medical Oncology-Research, University of Texas MD Anderson Cancer Center, Houston, TX USA; 15 Department of Medicine, Memorial Sloan Kettering Cancer Center, New York City, NY USA; 16 Weill Cornell Medical College, New York, NY USA; 17 Department of Medicine, Roswell Park Cancer Institute, Buffalo, NY USA; 18 Pediatrics, Human Oncology and Genetics, University of Wisconsin, Madison, WI USA; 19 UW Carbone Cancer Center, Madison, WI USA; 20 Johns Hopkins University School of Medicine, Baltimore, MD USA; 21 Center for Cancer Immune Therapy (CCIT), Department of Hematology, University Hospital Herlev, Herlev, Denmark; 22 Department of Immunology and Microbiology, University of Copenhagen, Herlev, Denmark; 23 Clinical Center, National Institutes of Health, Bethesda, MD USA; 24 Department of Surgical Oncology, The University of Texas MD Anderson Cancer Center, Houston, TX USA; 25 Medicine, Immunology and Dermatology Institute, University of Pittsburgh School of Medicine, Pittsburgh, PA USA; 26 Cancer Diagnosis Program, Division of Cancer Treatment and Diagnosis, NCI, NIH, Rockville, MD USA; 27 Istituto Nazionale Tumori di Napoli Fondazione “G. Pascale”, Via Mariano Semmola, 80131 Naples, Italy

**Keywords:** Melanoma, Immunotherapy, Cancer, Checkpoint blockade updates, Combination therapies, Biomarkers

## Abstract

Major advances have been made in the treatment of cancer with targeted therapy and immunotherapy; several FDA-approved agents with associated improvement of 1-year survival rates became available for stage IV melanoma patients. Before 2010, the 1-year survival were quite low, at 30%; in 2011, the rise to nearly 50% in the setting of treatment with Ipilimumab, and rise to 70% with BRAF inhibitor monotherapy in 2013 was observed. Even more impressive are 1-year survival rates considering combination strategies with both targeted therapy and immunotherapy, now exceeding 80%. Can we improve response rates even further, and bring these therapies to more patients? In fact, despite these advances, responses are heterogeneous and are not always durable. There is a critical need to better understand who will benefit from therapy, as well as proper timing, sequence and combination of different therapeutic agents. How can we better understand responses to therapy and optimize treatment regimens? The key to better understanding therapy and to optimizing responses is with insights gained from responses to targeted therapy and immunotherapy through translational research in human samples. Combination therapies including chemotherapy, radiotherapy, targeted therapy, electrochemotherapy with immunotherapy agents such as Immune Checkpoint Blockers are under investigation but there is much room for improvement. Adoptive T cell therapy including tumor infiltrating lymphocytes and chimeric antigen receptor modified T cells therapy is also efficacious in metastatic melanoma and outcome enhancement seem likely by improved homing capacity of chemokine receptor transduced T cells. Tumor infiltrating lymphocytes therapy is also efficacious in metastatic melanoma and outcome enhancement seem likely by improved homing capacity of chemokine receptor transduced T cells. Understanding the mechanisms behind the development of acquired resistance and tests for biomarkers for treatment decisions are also under study and will offer new opportunities for more efficient combination therapies. Knowledge of immunologic features of the tumor microenvironment associated with response and resistance will improve the identification of patients who will derive the most benefit from monotherapy and might reveal additional immunologic determinants that could be targeted in combination with checkpoint blockade. The future of advanced melanoma needs to involve education and trials, biobanks with a focus on primary tumors, bioinformatics and empowerment of patients and clinicians.

## Evolving topics in cancer immunotherapy

### Radiotherapy and immunotherapy

The first evidence that T cells contribute to the local (and possibly systemic) response to tumor-targeted radiotherapy (RT) was published over 30 years ago [1], but as only recently it was demonstrated in pre-clinical cancer models that the abscopal effect (i.e., tumor regression outside of the field of radiation following RT to one lesion) is immune-mediated [2]. Progress in understanding the complex molecular mechanisms that regulate T cell activation, migration to tumor site and effector functions within tumors has led to the identification of several mechanisms that prevent immune-mediated tumor rejection in most patients. Some of these mechanisms have been successfully targeted therapeutically by using antibodies blocking inhibitory immune checkpoint receptors such as cytotoxic T-lymphocyte associated protein 4 (CTLA-4) (e.g., ipilimumab) and programmed death 1 (PD-1) (e.g., nivolumab, pembrolizumab, atezolizumab) [3].

However, most patients do not respond to single agent’s therapy with immune checkpoint blockers (ICB). In this context, RT has been demonstrated to be a good combination partner for ICBs that increases responses against poorly immunogenic tumors in mice [4], and sometimes in patients [5, 6]. Proof-of-principle studies have demonstrated that RT can contribute at three levels to immune-mediated tumor rejection: (1) by generating anti-tumor T cells; (2) by overcoming T cell exclusion from the tumor; (3) by enhancing T cell-mediated recognition and killing of cancer cells that survive RT [7]. Thus, RT is under investigation as a modifier of the tumor microenvironment shifting so called “cold” tumors, which lack immune infiltrate (refractory to ICB), to so called “hot” tumors with lymphocyte infiltrate present (responsive to ICB).

Despite this experimental evidence, the promise of RT to convert the irradiated tumor into an in situ vaccine and elicit systemic anti-tumor immune responses capable of mediating abscopal effects (i.e., regression of metastases outside of the field of radiation) remains elusive. A number of active clinical trials combining RT with ICBs are ongoing: 18 trials testing radiotherapy with anti-CTLA-4, 51 trials testing radiotherapy with anti-PD-1/PD-L1, with radiotherapy regimens varying from 1.8 GyX28, to 3 GyX10, to stereotactic radiosurgery with single doses of 20 Gy or more [8].

Our group has investigated the impact of RT dose and fractionation on the ability of RT to synergize with ICBs and induce abscopal effects. We found that a hypofractionated regimen of 8GyX3 was effective while a single dose of 20 Gy was not [9]. In addition, we recently identified the mechanism underlying this difference in immunogenicity of different RT regimens. Hypo-fractionated RT induces cancer cell-intrinsic Interferon type I (IFN-I) pathway activation and production of IFNβ, which is required for optimal recruitment to the tumor of BATF3-dependent dendritic cells. Cancer cell expression of the cytoplasmic DNA sensor cGAS and its adaptor STING are required for RT-induced IFN-I production. Inducible knockdown of cGAS or STING in the irradiated tumor abrogated the induction of anti-tumor CD8 T cells by 8GyX3 RT and CTLA-4 blockade, and abolished the occurrence of abscopal effects. Finally, since cGAS is a sensor for double-stranded (ds) DNA, we investigated its presence in the cytoplasmic fraction of cancer cells irradiated with 8GyX3 or 20 Gy. These experiments revealed that RT induces the accumulation of dsDNA only in cancer cells treated with 8GyX3 but not 20 Gy [10]. In conclusion, the radiation dose and fractionation is a critical determinant of RT synergy with ICBs. Stimulation of cancer cell-intrinsic IFNβ production by RT is required to prime anti-tumor CD8 T cells to poorly immunogenic tumors. These findings have important implications for the choice of RT dose and fractionation when used in combination with ICBs.

Immunocytokines (ICs) are a class of molecules created by linking tumor-reactive monoclonal antibodies (mAbs) to cytokines that are able to activate immune cells. Tumor selective localization is provided by the ability of the mAb component to bind to molecules found on the tumor cell surface and molecules found selectively in the tumor microenvironment. In this way, the cytokine component of the immunocytokine is selectively localized to sites of the tumor and can activate immune cells with appropriate receptors for the cytokine.

It has been previously shown that an intratumoral (IT) injection of IC, which consists of an antitumor Ab specific to disialoganglioside (GD2) linked to interleukin (IL)-2, can serve as an in situ vaccine. It enhances local antitumor effects and can generate an adaptive T cell response directed against distant tumors. These in situ vaccine effects involve T cells as well as NK cells, and can result in T cell memory in melanoma and neuroblastoma (NBL) as demonstrated in preclinical studies. Preclinical studies have shown that tumor-reactive mAbs can mediate in vitro tumor destruction via antibody-dependent cellular cytotoxicity (ADCC). Based on these results clinical approach was developed at the Children’s Oncology Group (COG) administering this agent in individuals with smaller burden of cancer (non-bulky disease) [11]. This approach was tested clinically in the minimal disease setting in a pilot COG phase 1 trial for children with high risk NBL that were in remission after autologous hematopoietic stem cell transplantation (HSCT) (ASCT) but likely to relapse. To augment ADCC cytokines such as IL-2 to activate natural killer (NK) cells and granulocyte–macrophage colony stimulating factor (GM-CSF) to activate neutrophils/macrophages were incorporated. When this same regimen was moved into a large COG phase 3 trial with immunotherapy the treatment was statistically superior to the control treatment for both event-free survival (66% vs. 46% p = 0.01), and for overall survival (86% vs. 75% p = 0.02). These data suggest that other ADCC-mediating mAbs (i.e., rituximab, herceptin and erbitux) might be considered for trials in which high risk patients likely to relapse receive these mAbs in combination with agents known to activate ADCC (e.g., IL-2 + GM-CSF).

Because the efficacy of anti-GD2 mAb + cytokines in NBL trial was only 66% and NBL-free survival at 2 years further enhancement of the clinical potency of ADCC to obtain even better clinical results was undertaken [11]. The ICs were constructed by fusing the human IL-2 gene to the chimeric (ch) 14.18 or humanized (hu) 14.18 IgG1 genes to activate IL-2 receptor positive (IL-2R+) effector cells with a molecule that bridges them to tumor cells and then activates them. Indeed it was shown that these ICs activate GD2-specific tumor cell binding by IL-2R+ T cells and NK cells [11]. Ch14.18-IL-2 induces anti-melanoma activity in a SCID-xenograft model and in conventional mice bearing syngeneic tumors expressing GD2 (B78 melanoma); and anti-NBL activity in conventional mice bearing the GD2+ NXS2 NBL [11]. However, when IC (hu14.18-IL-2) was used clinically, anti-tumor activity is accompanied by dose-limiting IL-2-related toxicities suggesting the need to design the reagents and mouse models that better simulate the potential activity of IL-2-based on in vivo immunotherapy in patients [12].

As a single-agent immunotherapeutic approaches can have limited efficacy, combining two or more immunotherapeutic strategies can be synergistic in inducing antitumor effects. There is a growing enthusiasm for testing checkpoint blockade in combination with other approaches to augment immune-mediated antitumor effects. Recently synergistic effect of the combination of anti-CTLA-4 mAb blockage and intratumor (IT) administration of the IC on smaller tumors (day-7 B78, < 50 mm^3^) was demonstrated although the treatment was less efficacious on larger tumors (day 12, B78 tumors) [13]. Recent studies of mAb/cytokine-based immunotherapies for solid tumors have shown IT-IC is more effective for measurable mouse tumors [13, 14] and that IV treatment (mAb or IC) can be effective in minimal residual disease (MRD) setting (COG studies) [15].

However, combination with immunomodulatory, radiation and IT hu14.18-IL-2 administration results in cure of most large tumors (5-week, 200 mm^3^, B78) [14]. Results show that combining RT and IT-IC in murine tumor models can eradicate large tumors and metastases. This suggests that in situ vaccination effect can be enhanced by T cell checkpoint blockade, with implications for clinical evaluation. Preclinical data demonstrate that IT-IC + RT (and anti-CTLA-4) activates innate and adaptive immunity, overcomes tumor induced immune suppression, and facilitates use of existing tumors as in situ vaccines.

The following data support the strategy of increasing the efficacy of the IC vaccine via IT delivery: (1) IT-IC causes much higher levels of IC in the injected tumor than IV-IC; (2) Greater IC levels in the tumor enhance NK infiltration into the tumor (via FcRs and IL-2Rs), leading to greater ADCC and greater tumor destruction, even of larger macroscopic lesions that are unresponsive to IV IC delivery; (3) Some of the IC injected IT circulates systemically (via lymphatics and blood vessels), enabling IC delivery to distant sites as effectively as when IC is given IV (possibly with a better PK profile); (4) The IC-facilitated response within the tumor may attract other effector cells (T cells and macrophages) to the tumor site (or to draining lymph nodes), leading to T cell sensitization; (5) The vaccine-like effect resulting in tumor-specific T cell reactivity may impact on distant sites of micrometastatic disease (and prevent subsequent growth upon experimental tumor re-challenge); and (6) Combining IC with other treatments that cause localized tumor damage (without local immune suppression), should synergistically augment the antitumor activity of the IC.

### Cancer vaccines in the era of checkpoint blockade

A cancer vaccine is a preparation of a tumor antigen that upon administration stimulates antibody production or cellular anti-tumor immunity. In fact, it is known that T cells can mediate remarkable tumor regressions including complete cure in patients with metastatic cancer. The mutanome, the collective genetic alterations in an individual’s cancer cells, encodes peptides (M-peptides) that can function as unique therapeutic targets as neoantigens.

M-peptides in individual patients can be identified by next-generation sequencing and computational algorithms guided approaches for T cell epitope prediction. Although there is a correlation between peptide binding affinity for MHC class I and II and immunogenicity, other factors also contribute. For example, sufficient affinity of the interaction between MHC-bound mutated peptide and the T-cell receptor (TCR) is essential for the recognition of the mutated peptide as ‘foreign’. The identification of epitopes that drive the immune response in cancer is essential to the understanding and manipulation of CD8 T-cell immune responses for clinical benefit. Recent studies in mice and humans have suggested that tumor-specific mutations may have a key role in shaping the anti-tumor response; but their identification remains a challenge [16]. To be fully useful in the clinic, it will be necessary to rely on computational predictions, including structural features of the MHC I-haplotype-specific and whole-exome/transcriptome sequencing of a patient’s tumor, which is beginning to be routinely determined.

In 2011, Schwartzentruber et al. found that combining a melanoma vaccine with IL-2 [high-dose IL-2 ± gp100 peptide in incomplete freund adjuvant (IFA), i.e., water-in-oil emulsion], improved the response rate and progression-free survival in patients with advanced melanoma, versus IL-2 alone [17]. Currently there are 369 open studies using cancer vaccines in the USA only, most with little or no evidence of tumor regression. Limited tumor regression following treatment with vaccines could also be due to immunosuppressive tumor microenvironment even in the presence of increased frequency of cancer-specific T cells. These T cells in the tumor microenvironment are likely those that are not proinflammatory, or with poor T cell effector function/wrong phenotype. Other tumor phenotypes that account for most of nonresponders are due to few T cells or poor T cell trafficking to tumor. Insufficient spontaneous T cell reactivity and/or lacking immune cell infiltration to tumor site could be one of the limitations of effective anti-tumor response [18]. Such tumor-specific T cell responses could be induced through anti-cancer vaccination, but despite great success in animal models, only a few of many cancer vaccine trials have demonstrated robust clinical benefit.

As vaccine adjuvants determine the type and magnitude of the T cell response after vaccination one possible explanation for the lack of efficacy of vaccine therapy in humans is the use of safe, but very weak vaccine adjuvants in clinical trials as opposed to the use of potent, effective vaccine adjuvants in animal models [18]. Vaccine adjuvants for peptide-based cancer vaccines can function as an antigen depot for prolonged release, can protect antigen from degradation, can increase antigen uptake by antigen presenting cells (APCs) or can induce a pro-inflammatory/pro-immunogenic milieu.

Persisting peptide/IFA vaccine depots can induce specific T cell sequestration, dysfunction and deletion at vaccination sites, possibly explaining lack of synergy between gp100 peptide vaccination and ipilimumab in patients with melanoma [19]. On the contrary, short-lived formulations can overcome these limitations and result in greater therapeutic efficacy of peptide-based cancer vaccines [20].

Recently, Hailemichael et al. demonstrated that IFA-based vaccination does not synergize with anti-CTLA-4 therapy and that IFA-based vaccination sequesters T cells induced by anti-CTLA-4 therapy. More promising results were obtained from virus-based vaccination that synergizes with anti-CTLA-4 therapy and water-based peptide vaccination that synergizes with anti-PD-1 therapy (Hailemichael et al., in press). Given that the adjuvant choice determines T cell response to cancer vaccine, molecular adjuvants like TLR agonist, CD40 agonists or cytokine are investigated.

IT immunotherapy can be an alternative to inefficient systemic cancer vaccines. Such therapy could empower the immune system to mount T cell responses against various immunogenic tumor-associated antigens. To mediate systemic tumor regression, intratumoral (IT) immunotherapy must generate systemic T cell responses that can target distant metastases beyond the initially treated tumor mass [21].

Intratumoral (IT) treatment with 3M-052 (an injectable, tissue-retained TLR 7/8 agonist) was found to be a promising approach for the treatment of cancer thus establishing a rational strategy for combination therapy with IT, tissue-retained TLR7/8 agonist and checkpoint blockade in metastatic cancer [22]. IT administration of 3M-052 generated systemic antitumor immunity, and sensitized both injected an uninjected wild-type B16.F10 melanomas to checkpoint blockade therapy with anti-CTLA-4 and anti-PD-L1 antibodies, even when checkpoint blockade alone was ineffective [22].

In conclusion, cancer vaccines can have clinical impact by synergizing with checkpoint blockade. To induce better T cell responses and clinical impact, relevant factors are formulation (linked to possible T cell sequestration), addition of multiple immunomodulators (cytokines, TLR agonists), combination with checkpoint blockade and use of intratumoral immunotherapy as a vaccine strategy need to be considered.

### Tumor infiltrating lymphocytes (TIL) therapy in melanoma

Adoptive T cell therapy (ACT) with autologous tumor infiltrating lymphocytes (TILs) is an effective treatment for patients with metastatic melanoma. TILs that are expanded in vitro, and reinfused in conjunction with IL-2 following a lymphodepletion has been a method for treatment of heavily pretreated cancer patients [23]. ATC treatment shows objective responses in about 50% of patients in clinical trials, with a 20% of complete responses. TILs are mainly CD T-cell-based cultures and better quality of TILs are associated with better clinical outcomes including overall survival. Higher T cell infiltrate in tumors could be accomplished by vaccination, target therapy (e.g., BRAF) and the exercise.

However, TIL therapy infusion products lack strong predictive markers and the limitation of the optimal responses include inability of T cells to infiltrate the tumor site, immunosuppressive environment, and the quality and quantity of TILs. Efforts are ongoing to generate more potent TILs or to improve intratumoral infiltration in patients including co-stimulation through the 4-1BB/CD137 antibody which increases the CD8+ T cells frequency. The use of K562− derived artificial antigen presenting cells (APCs), which act as a feeder cells for T-lymphocytes expansion is also used. Among methods to improve adoptive T-cell therapy, the administration of checkpoint inhibitory antibodies activating T cells or depletion of myeloid derived suppressor cells (MDSC) to reduce immunosuppression are proposed. Another method is re-stimulation of the injected TILs with a tumor vaccine to improve life-span of the antigen specific T cells. Other strategies to counterbalance tumor-driven immune dysfunction are reversing the oxidative stress in cancer patients by the administration of histamine and antioxidants (e.g., vitamin E).

Exercise can exert a great anticancer effect [24–26]. Regular exercise delay progression and growth of tumors in numerous experimental models [27, 28]. Furthermore, therapeutic effect of exercise on cancer is demonstrated in cancer patients, for example decreased tumor progression in prostate cancer [26]. Multiple studies (n = 88) reported on associations between physical activity after diagnosis and prognosis among cancer survivors showing an impact of exercise on cancer survival. Unfortunately, there are differences in the models and the type of physical intervention which make difficult comparisons with cancer survival. Mechanism(s) for the effect of physical activity in cancer remain unknown although dietary and hormonal factors have been postulated (e.g., insulin and insulin-like growth factor, inflammatory markers).

Recent findings demonstrated that voluntary wheel running significantly reduces tumor incidence and growth in various experimental tumor models [29]. Exercise, cancer, and immunity are linked as exercise decreases tumor incidence and growth by over 60% across several mouse tumor models. Exercise control of tumor growth is mediated through a direct regulation of NK cell mobilization and trafficking. Mechanisms involve epinephrine-dependent mobilization of NK cells to the circulation and IL-6-dependent redistribution to the tumors. In particular, exercise increases NK cell infiltration into tumor site, thereby controlling tumor growth. Epinephrine mobilizes NK cells and blunts the tumor suppression. In addition, exercise-induced muscle-derived IL-6 is involved in NK cell redistribution [29]. For example, subcutaneous B16F10 melanoma model in female mice demonstrated that 4 weeks of wheel running prior to tumor cell inoculation reduced tumor growth by 61% (p < 0.01). These findings indicate that exercise might deliver a therapeutic effect with increased immune cell infiltration and generation of an inflammatory intratumoral environment [29].

In conclusion, exercise, may lead to mobilization and tumor infiltration of T and NK cells animal models. Although the mechanism of increase in physical activity on survival and recurrence in cancer patients are not established as the randomized controlled trials are needed to generate definite data. However, it can have positive impact on efficacy of immune therapy, including ACT such as TILs or other types of ACT (e.g., CARs, genetically modified T cells etc.) as demonstrated in animal models.

## System biology session: molecular

### MicroRNAs and drug resistance to melanoma

Drug resistance is major issue in medical oncology because its development limits the long-term efficacy of current cancer therapies. Understanding the mechanisms behind the development of acquired resistance will offer new opportunities for more efficient combination therapies. Some concepts have been demonstrated.

Melanoma patients bearing BRAF V600 mutation benefit from therapy with BRAF inhibitors [30]. Short term (6 months) benefit of BRAFi therapy was demonstrated [31]. A major issue in the treatment of BRAF mutated metastatic melanoma is the disease relapse caused by emergence of drug resistance. In most cases BRAFi resistant melanoma bear mutations or molecular aberrations reactivating the MAPK pathway [32]. Frequent reactivation of MEK in BRAFi resistant tumors led to the development of BRAFi + MEKi combination therapies. Combination therapy with BRAF and MEK inhibitors improves survival but is unable to prevent disease relapse [33]. In tumors resistant to BRAFi + MEKi dual therapy the same type mutations are found. Therefore, hitting hard two different targets in the same pathway doesn’t solve the problem and the identification of additional mechanisms responsible for drug resistance is an unmet need.

In a significant percent of cases (26%) no new mutations that constitute the basis of resistance could be identified. Other types of molecular changes than mutations as adaptive mechanisms can contribute to drug resistance in melanoma [34, 35]. MicroRNAs that are important multifunctional post-transcriptional modulators of gene expression affect multitude of cellular pathways. MicroRNAs also play a key-role in various progression-related and invasive properties of human cancers. In addition, there is a growing evidences suggesting miRNAs as key factors controlling the emergence of drug resistance [36]. MicroRNAs (miRNAs) are small noncoding RNAs that modulate gene expression by mRNA silencing or degradation, which usually have pleiotropic effects because of their ability to target simultaneously multiple mRNAs. The first example of a miRNA-dependent mechanism of drug resistance in BRAF mutated melanoma focused on a previously poorly characterized miRNA, miR-579-3p. Main findings were: (1) low expression of miR-579-3p is a negative prognostic factor correlating with poor survival; (2) expression levels of miR-579-3p decrease during melanoma progression i.e., from nevi to stage III/IV melanoma; (3) miR-579-3p acts as oncosuppressor by targeting the 3′ untranslated region (3′UTR) of two oncoproteins (BRAF and an E3 ubiquitin protein ligase, MDM2); (4) moreover miR-579-3p ectopic expression impairs the establishment of drug resistance in human melanoma cells; and (5) miR-579-3p is strongly down-regulated in matched tumor samples from patients before and after the development of resistance to targeted therapies [37] and was also identified in cell lines resistant to BRAF/MEK inhibitors.

Recently, the assessment of changes in the whole miRNAome profile during the development of drug resistance in vitro in two different BRAF-mutated melanoma cell lines was performed by the Nanostring™ platform (Fattore et al., unpublished). Data revealed a stepwise deregulation of a growing number of miRNAs. Deregulated miRNAs of resistant melanoma cells mostly impact on pro-inflammatory and pro-angiogenetic pathways. Besides, conditioned medium from drug resistant WM266 cells triggers directional cell migration, promotes angiogenesis, and its effect is fully inhibited by VEGF antagonists (Fattore et al. unpublished). Per other findings, anti-PD-1 resistant tumors display a transcriptional signature (IPRES) resembling that of MAPK-resistant melanomas which is at the basis of cross-resistance. If a similar network of miRNAs contributes to cross-resistance between MAPKi and checkpoint inhibitors is still to be investigated.

### Melanoma mutations and ‘precision medicine’

The notion of “precision medicine” is to include molecular markers based on information derived from genetics, epigenetics, gene expression and proteomics data to diagnose and treat patients. This subject is not new as reflected by over 900 publications since 2009. Indeed, several institutions already searched for a panel of “actionable” mutations (in addition to BRAF and NRAS), for targeted therapy. These include MSK-IMPACT, FoundationOne, and IBM Watson Genomics. Ideally, NextGen Sequencing will be used in the near future to distinguish primary melanomas from benign nevi, to identify tumor heterogeneity, new targets for therapy, interactions of tumor cells with the microenvironment, the presence of neoantigens, and to monitor tumor load, just to name a few. However, the main obstacles to achieve these goals are speed of getting the results and the high cost of the tests. One way to alleviate the problem is the use MelArray (Melanoma Targeted Sequencing) designed by Dr. Michael Krauthammer from Yale University in collaboration with Dummer Reinhard and Levesque at Dermatology, University of Zurich. This array is composed of 190 melanoma mutant genes, TERT promoter, and 28 introns across eight genes to identify fusion genes such as BRAF, RAF1, ALK, MAP3K8, MET, NTRK1, PRKAR1A, and ROS1. The array has reasonable sequencing costs; currently about five time lower than whole exome sequencing.

A group of investigators at Yale University tested the MelArray on several tumors and identified the critical mutations and genomic aberrations. An interesting case was the discovery of the molecular basis of tumor heterogeneity in one patient. The four portions of the tumor, all isolated at the same time, displayed NRAS^Q61R^, IDH1^R132C^, DDX3X^P274L^, ASPM^R1763K^ and TERT 5′Flank SNV, but were different in CTNNB1 mutations. We identified CTNNB1^S45Y^, CTNNB1^S45*^, and wild-type gene in two other sites. Interestingly, the DDX3X, (DEAD-box helicase 3, X-linked) might render the tumor susceptible to RK-33 [38]. However, it is likely that we need to continue performing WES to find out the total number of mutations and the neoantigens.

Other discoveries are fusion genes in wild type BRAF and NRAS melanomas, which include PDE8A-RAF1, PDE4DIP-BRAF and NFIA-BRAF. We demonstrated that PDE8A-RAF1 is a transforming gene that activates the MAPK and confers growth factor independence in mouse melanocytes. Melanoma cells with PDE8A-RAF1 or PDE4DIP-BRAF were resistant to vemurafenib but highly sensitive to selumetinib and SCH772984 (ATP independent MEK1, 2 and ERK inhibitors, respectively).

Because molecular analyses generate massive amounts of data, there is a need to partner with computational biology cores. For example, converting the variant call format (VCF) to actual mutations can be a long process. We tested the IBM Watson Genomics supports and received within minutes the genetic alterations of the tumor as well as options for targeted therapy.

In addition to mutations and genomic aberrations, we need to analyze gene expression that can help in predicting response to therapy, especially immunotherapy. RNA sequencing of bulk tumors can provide information regarding the composition of TIL subsets in the tumor microenvironment including IFNγ and IFN pathway genes, neoantigen load, and expression of PD-L1 in the tumor tissue and micro-environment. Several programs were developed to profile leukocyte composition directly from RNA sequencing data, and one of them is CIBERSORT (cell type identification by estimating relative subsets of known RNA Transcripts) [39].

In conclusion, a list of ‘omics’ and functional screening test should include but is not limited to:

1. Next generation sequencing (NGS): routine analysis of the molecular changes in the tumor(s) in each patient; provides therapy targets and biomarkers as well as biological input.
2. RNAseq: TILs to be profiled with increasingly high resolution and accuracy directly from RNA mixtures of bulk tumor samples.
3. Non-invasive test, such as CAPP-Seq (cancer personalized profiling by deep sequencing of circulating tumor DNA) [40].
4. Proteomics analyses in tissue and blood.
5. Functional studies, in vitro and in vivo drug response to determine the effects of genomic alterations.

### Biomarkers for treatment decisions

Biomarkers have opened the paradigm of ‘precision medicine’ by incorporating biomarkers for risk assessment and screening (prognostic); at diagnosis, when markers can assist with staging, grading (diagnostic), and therapy selection (predictive of response); and to select additional therapy or monitor for recurrent disease in clinical management of cancer patients.

Biomarkers such as mutations (NRAS, c-Kit, BRAF) are already critical for proper management of advanced melanoma patients. Based on the recent data of anti-PD-1 treatment efficacy in PD-L1-positive advanced melanoma, PD-L1 expression may reflect the presence of T cells secreting IFNγ in the tumor microenvironment [41]. PD-L1 expression in tumors correlates with the presence of TILs, since IFNγ production by TILs can induce expression of PD-L1. Therefore, this category of tumors is likely to respond to TIL targeted therapy unlike tumors who do not have T cell infiltrate.

Treatment of systemic metastatic disease (stage IV) involves new therapeutic strategies. Immunotherapy, that utilizes antibodies that bind to checkpoint inhibitory receptor of T-cells such as CTLA-4 blocking agent ipilimumab; the anti-PD-1 antibodies, such as nivolumab and pembrolizumab have already demonstrated impressive efficacy [41]. Targeted therapy agents such as selective BRAF inhibitors including vemurafenib, encorafenib and dabrafenib used alone and/or in combination with MEK inhibitors such as binimetinib, cobimetinib and trametinib, have also demonstrated impressive antitumor activity. Therefore, immunotherapy and kinase inhibitors have become the backbone of systemic therapy in melanoma.

The field of genetics has made advances in recent years and methods to deliver and share genomic information for clinical care across different tumor types become available. For example, the Foundation of Medicine T5 Gene Panel allows Targeted DNA Sequencing of 300 genes across all four classes of genomic alterations to help understand the genomic makeup of a patient’s tumor. In this assay, next-generation sequencing (NGS) is used to analyze cancer specimens for all four classes of genomic alterations (base substitutions, insertions and deletions, copy number alterations, and rearrangements). The targeted sequencing focuses on annotated and actionable mutational findings. In addition, high sequencing depth also overcomes contamination with normal tissue and allows for the detection of mutations at subclonal level. Genomic profile analysis using T5 Gene Panel demonstrated that the frequency of genetic alterations of CDKN2A and CDKN2B pathway correlated with short (< 3.5 months) and longer progression free survival (PFS) in melanoma patients. This cell cycle pathway was frequently altered and it is driven primarily by CDKN2A alterations. As far as HGF/MET alterations are concerned several patients had amplification of genes on the 7q chromosome. These patients tended to have a shorter PFS than the BRAF-mutant population and seven patients exhibited MET (n = 4) and/or HGF (n = 6) amplifications, with co-amplification occurring in 3 of 6 patients. PFS was < 3.5 months for 3 of 4 patients with the MET amplification and 5 of 6 patients with the HGF amplification (van Herpen et al., submitted).

Furthermore, HGF expression may contribute to therapeutic resistance in BRAF-mutant melanoma. A recent study in patients with BRAF-mutant melanoma reported correlation between HGF expression by stromal cells and innate resistance to BRAF inhibitor treatment. Response to treatment (BRAFi ± MEKi) was significantly lower in patients with stromal HGF expression than in patients without HGF expression (p < 0.05) [42]. Treatment with a BRAF inhibitor increased stromal HGF expression in several patients. Besides, in BRAF-mutant cell lines treated with a BRAFi and HGF, HGF induced sustained activation of ERK and AKT, which was more pronounced under BRAF inhibition than under MEK inhibition. These results suggest that genetic alterations leading to dysregulation of the MAPK pathway (e.g., HGF amplification) could contribute to resistance to BRAFi therapy in patients with BRAF-mutant melanoma [42]. These data support hypothesis that stromal cells might confer innate resistance on cancer cells and cancer drugs.

Oncoprotein-targeted drugs hold enormous promise for the future of cancer treatment. However, complete clinical responses are rare, suggesting that mechanisms exist to render a substantial proportion of tumor cells resistant to treatment. Biomarker analysis of tumors following MAPK pathway inhibition of MEK by binimetinib demonstrated that, the tumor genetic landscape was concordant with what was reported in the TCGA melanoma database. Driven primarily by CDKN2A alterations, the cell cycle pathway was frequently altered in patients with the BRAF mutation. Several patients had amplification of genes on the q-arm of chromosome 7. Three of 7 of these patients had a PFS that was lower than the median PFS for the BRAF-mutant population, suggesting that a gene or genes on 7q may promote MEKi resistance. Genes of high interest located on 7q include the HGF/MET pair, as well as BRAF itself (van Herpen et al., submitted).

Another subtype of melanoma is characterized by NRAS mutation that occurs in ~ 20% of patients. Despite significant efforts to develop drug specific targeting NRAS there are no specific therapies for NRAS mutated melanoma. Despite emergence of immunotherapies as effective treatments in melanoma, there is still a need to develop therapies for these patients, particularly after failure of NEMO open-label phase 3 study of binimetinib vs dacarbazine in patients with advanced unresectable/metastatic cutaneous NRAS-mutant melanoma who were previously untreated or had progressed on/after and who showed improvement in PFS [43].

Pre-clinical data on the BRAF kinase inhibitor vemurafenib showed response rates of more than 50% in patients with metastatic melanoma with the BRAF V600E mutation. The next phase 3 randomized clinical trial comparing vemurafenib with dacarbazine in 675 patients with previously untreated, metastatic melanoma with the BRAF V600E mutation resulted in improved rates of overall and progression-free survival (relative reduction of 63% in the risk of death and of 74% in the risk of either death or disease progression, as compared with dacarbazine (p < 0.001) [31]. Vemurafenib is a potent inhibitor of V600 mutant BRAF but cutaneous side effects are frequent: peculiar cutaneous profile involving epidermis and adnexa overlaps with the cutaneous manifestations of genetic diseases characterized by activating germ line mutations of RAS (RASopathy) [44].

The mutational landscape of melanoma was studied by sequencing the exomes of 147 melanomas: among the genes, the PPP6C that encodes a serine/threonine phosphatase was found exclusively in tumors with mutations in BRAF/NRAS. This activating mutation changes Pro29 to serine (RAC1^P29S^) promoting melanocyte proliferation and migration, with possible therapeutic potential [45].

Furthermore, sequencing allows to investigate acquired chemotherapeutic resistance of cancer: the sequenced exomes of 27 lesions from three metastatic melanoma patients treated with targeted or non-targeted inhibitors showed that BRAF and NRAS co-mutations are not mutually exclusive. However, the sole finding of double mutated cells in a resistant tumor is not sufficient to determine follow-up therapy; these findings demonstrate that, in order to target the large pool of heterogeneous cells in a patient, the combinational therapy targeting different pathways is required [46].

In conclusion, immune checkpoint inhibitors, such as anti-CTLA-4 blockade that activates T cells and enables them to destroy tumor cells, are effective cancer treatments, but molecular determinants of clinical benefit are not defined. Whole-exome sequencing of tumor tissue from melanoma patients treated with ipilimumab or tremelimumab including mutational profiling and HLA analysis identified the presence of specific tumor neoantigens that might explain the therapeutic benefit. This and other published study illustrated the importance of tumor genetics in defining the basis of the clinical benefit from immunotherapy including CTLA-4 and PD-1 pathway blockade [47].

## Combination strategy session

### Overcoming radiation-induced barriers to an immune response

Radiation therapy is a highly effective local treatment for cancer. Over the past 50 years, sporadic events of tumor regression in un-irradiated fields, at distant metastatic sites, known as abscopal effects, have been observed: a total of 46 reported cases have been identified from 1969 to 2014 with median radiation dose of 31 Gy, median follow-up of 17.5 months, and median documented time to notice the abscopal effect was 2 months [48].

The abscopal effect of radiation has more recently been demonstrated to be a result of an antitumor immune response induced by radiation effects in the irradiated tumor. The rarity of abscopal effects is a consequence of the fact that at the time of metastasis cancer is associated with a profound and tumor-specific immune-suppressive status. Modern immunotherapy, has offered the potential for a recovery of an immune response and a synergy with immune activation by radiation. The combination with immunotherapy has defined a novel role for radiotherapy in systemic disease.

Barriers to the potential of radiation to convert a tumor into an in situ vaccine have emerged beyond the pre-existing immunosuppressive microenvironment of established tumors. Radiotherapy itself also induces some immunosuppressive signals, within and beyond the irradiated field. Some trials are ongoing to overcome radiation-induced immunosuppression. For instance, counter-acting RT-induced immune-suppression mediated by adenosine, with effect on dendritic cells (DC) maturation and T cells recruitment was documented in several trials (MEDI9447; NCT02503774; NCT01283594). Recent findings demonstrate in murine models that CD73-blockade reduces radiation-mediated T regulatory cells (Tregs) infiltration while promoting CD8+ T cell infiltration and combined RT and anti-CD73 treatment delays tumor progression and prolongs survival. These data suggest that targeted CD73 therapy helps radiotherapy by enhancing the adaptive immune response machinery, which may increase the function of tumor-infiltrating T lymphocytes, and subsequently lead to improved survival in cancer patients.

Another approach regards overcoming TGFβ activation by radiation-induced ROS, a mechanism that hinders priming of anti-tumor T cells. Vanpouille-Box et al. demonstrated that antibody-mediated TGFβ neutralization during radiation therapy effectively generates CD8+ T cell responses to multiple endogenous tumor antigens in poorly immunogenic mouse carcinomas. Generated T cells were effective at mediating regression of irradiated tumors and non-irradiated lung metastases or synchronous tumors (abscopal effect). Gene signatures associated with IFNγ and immune-mediated rejection were detected in tumors treated with radiation therapy and TGFβ blockade in combination but not as single agents. However, up-regulation of programmed death ligand-1 and -2 (PD-L1 and 2) in neoplastic and myeloid cells and PD-1 on intratumoral T cells limited the persistence of tumor rejection, resulting in rapid recurrence. Addition of anti-PD-1 antibodies enhanced the immune response and extended survival of the animals treated with radiation and TGFβ blockade. Thus, TGFβ is a fundamental regulator of radiation therapy ability to generate an in situ tumor vaccine and its systemic effects are enhanced by PD-1 blockade. The combination of local radiation therapy with TGFβ neutralization and PD-1 blockade offers a novel individualized strategy for vaccinating patients against their tumors [49].

Tumor-infiltrating myeloid cells (TIM), including CD11b (Integrin subunit alpha, ITGAM)þF4/80 (EMR1)b protein positive tumor associated macrophages, and CD11bþGr-1 (LY6G)þ myeloid-derived suppressor cells (MDSC) respond to cancer-related stresses and promote tumor angiogenesis, tissue remodeling, and immunosuppression. While the role of myeloid derived suppressor cells is complex, radiation increases both macrophages and MDSC. Enhanced macrophage migration induced by conditioned media from irradiated tumor cells was completely blocked by a selective inhibitor of CSF1R. Mechanistic investigations revealed the recruitment of the DNA damage-induced kinase ABL1 into the nucleus where it bound the CSF1 gene promoter resulting in CSF1 gene transcription. When added to radiotherapy, a selective inhibitor of CSF1R suppressed tumor growth more effectively than irradiation alone. The CSF1/CSF1R signaling to recruit TIMs likely limits the efficacy of radiotherapy. Thus, CSF1 inhibitors should be evaluated in clinical trials in combination with radiotherapy as a strategy to improve outcomes [50].

Investigations to define the optimal dose, fractionation and field size of radiotherapy when combined with immunotherapy are ongoing. When combined RT with concurrent chemotherapy, radiation can cause severe treatment-related lymphopenia (TRL) (< 500 cells/mm^3^) that is associated with reduced survival. Severe TRL was observed in more than 40% of glioma patients 2 months after initiating chemo-radiation, an effect that was independently associated with shorter survival from tumor progression [51]. Yovino et al. modeled these effects in an attempt to predict the consequences of radiation-related variables, such as the number of fractions, the field size and the dose rate on circulating lymphocytes [52]. The model proposed is based on the assumption that a single radiation fraction delivers 0.5 Gy to 5% of circulating cells. Consequently, 99% of circulating blood would receive ≥ 0.5 Gy after 30 daily fractions. Reducing the number of fractions and the field size significantly decreases the exposure of circulating mononuclear cells during treatment [52]. Confirming this model, a retrospective study in locally advanced pancreatic cancer demonstrated significantly less severe radiation-induced lymphopenia from stereotactic body radiation than standard radiotherapy, that utilizes larger fields and more fractions of radiation [53]. Novel approaches are needed to limit radiation to circulating lymphocytes given the association of lymphopenia with poorer survival in patients.

In conclusion, radiotherapy induces both pro-immunogenic and immunosuppressive effects. RT reduces circulating lymphocytes rendering blood an “organ at risk”, especially for combination with immunotherapy. Multiple immunotherapy strategies may be required to circumvent both pre-existing and RT-induced immune-suppression with a strategy that combines hypo-fractionated, short courses of RT to small targets likely to be key to the success of RT + immunotherapy.

### Where are we really with clinical trials of combination immunotherapy?

There are two ways to think about combination immunotherapy:

1. Indirect methods: eradicate the tumor with immunogenic stimulus for example including radiotherapy, chemotherapy, oncolytic viral therapy or targeted therapy.
2. Direct methods include activating T cells through agonistic or antagonistic antibodies through co-stimulatory targets or turning off inhibitory receptors on T cells, respectively.

There are several targets with emerging data in melanoma, and this report selects a few for discussion.

OX40 is a co-stimulatory receptor that can potentiate T cell receptor signaling on the surface of T lymphocytes. In an OX40 phase I Trial, a mouse mAb that agonizes human OX40 signaling in patients with advanced cancer showed an acceptable toxicity profile and regression of at least one metastatic lesion in 12/30 patients [54]. Maximum tolerated dose was not reached (some fevers/chills, rash, fatigue, arthralgias); tumor regressions without formal RECIST partial responses; two patients with melanoma had mixed responses [54]. Phase I investigations of OX40 agonists are ongoing in multiple solid tumors and in combination with PD-1 and PD-L1/2 partial responses (RCC and UCC) were reported in 2016 [55].

Other co-stimulatory agonist drugs have been clinically evaluated in early phase trials with some early activity such as urelumab and utomilumab targeting activating receptor CD137/4-1BB. Ongoing studies in combination with PD-1: utomilumab plus pembrolizumab have also been conducted with results indicating that this combination is well tolerated with some responses being seen in solid tumors [56].

Another strategy involves blocking an intratumoral enzyme “checkpoint” called indoleamine 2,3 dioxygenase (IDO) a tryptophan-catabolizing enzyme that induces immune tolerance by T-cell suppression. IDO depletes tryptophan and produces toxic kynurenine. Epacadostat is an oral, potent, selective inhibitor of IDO1 and preliminary results from ongoing study of epacadostat with pembrolizumab showed promising clinical activity and acceptable safety profile. A dose expansion (epac 50, 100, and 300 mg BID + pembrolizumab 200 mg IV Q3 W) was then implemented (n = 22), showing a ORR in 19 untreated patients equal to 58%, and median PFS has not been reached [57]. Epacadostat is now in phase 3 study with pembrolizumab.

Combinations have been studied based on preclinical rationale and clinical evidence for efficacy as single agents. Early phase combination dose-finding studies and then ultimately randomized studies with an overall survival endpoint have been then implemented. Ipilimumab and GM-CSF combination has better overall survival than ipilimumab alone (HR 0.64 for OS) in a phase 2 study [58]. In a 2-year assessment of the phase 2 CheckMate-069 trial, the 2-year OS rate with the combination of nivolumab + ipilimumab was 69% compared with 53% for ipilimumab alone (many patients crossed over to nivolumab), for patients with BRAF wild-type melanoma [59]. The median OS among patients was not been reached with the combination regimen and was 24.8 months with ipilimumab monotherapy (HR, 0.58, 95% CI 0.31–1.08). In the overall study population, the 2-year OS rate was 64% with the combination compared with 54% for ipilimumab alone (HR, 0.74; 95% CI 0.43–1.26). The median OS at 2 years in patients randomized to either the combination or monotherapy has not been reached [60].

However, demonstrating overall survival benefits in randomized trials will be increasingly difficult due to the limited number of patients with melanoma available for trials and higher landmark overall survival metrics. New ways to test combinations are required. Should we add combinations to PD-1 non-responders or start combinations for “biomarker unfavorable” patients? Multiple combinations are partnering with PD-1, but should we reconsider ipilimumab combinations in the PD-1 refractory setting?

Neoadjuvant Trials are also being conducted. Their advantages are quick interpretation of drug effect, and they have pre-and post-treatment tissue available for pharmacodynamic analyses. Whether effects on macrometastatic disease in the neoadjuvant setting resemble efficacy against micrometastases in the neoadjuvant setting remain unknown. There is also the question of toxicity of systemic therapy in patients otherwise cured by surgery alone.

In conclusion, combinations are now testing multiple T cell regulators, intratumoral “checkpoints”, and standard anticancer agents. Reconsidering clinical trial endpoints other than overall survival will be needed. Neoadjuvant studies may offer unique opportunities.

### Sequencing and combinations of checkpoint inhibitors with targeted agents in melanoma

Immunotherapy has been a highly promising approach of melanoma treatment. Targeting BRAF mutation in patients harboring mutated gene has been shown to be also highly efficacious. Dual BRAF and MEK inhibition is associated with high response rates and median PFS (mPFS) of 9–12 months and superior survival compared to single-agent BRAF inhibitors (BRAFi) [61–63].

Comparison of systemic therapy for advanced (unresectable Stage III or Stage IV) melanoma reported in Table 1 shows similar efficacy in the median patient response, PFS and survival in selected group of patients.

**Table 1:** Comparison of systemic therapy for advanced (unresectable stage III or stage IV) melanoma

Treatment	RR (%)	PFS (med), months	OS (med/2-years)
Single-agent BRAFi	50	6–8	18.7 months/~ 40%
Combo BRAFi and MEKi	65–70	9–12	15 months/~ 50%
Ipilimumab	10	2–3	12 months/~ 30% (20% 5 year survival)
Anti-PD-1 mAb (nivolumab or pembrolizumab)	25–45	~ 6	18–24 months/~ 50%
Combo ipilimumab and nivolumab	~ 60	11–12	Unk./~ 64%

**Fig. 1:**
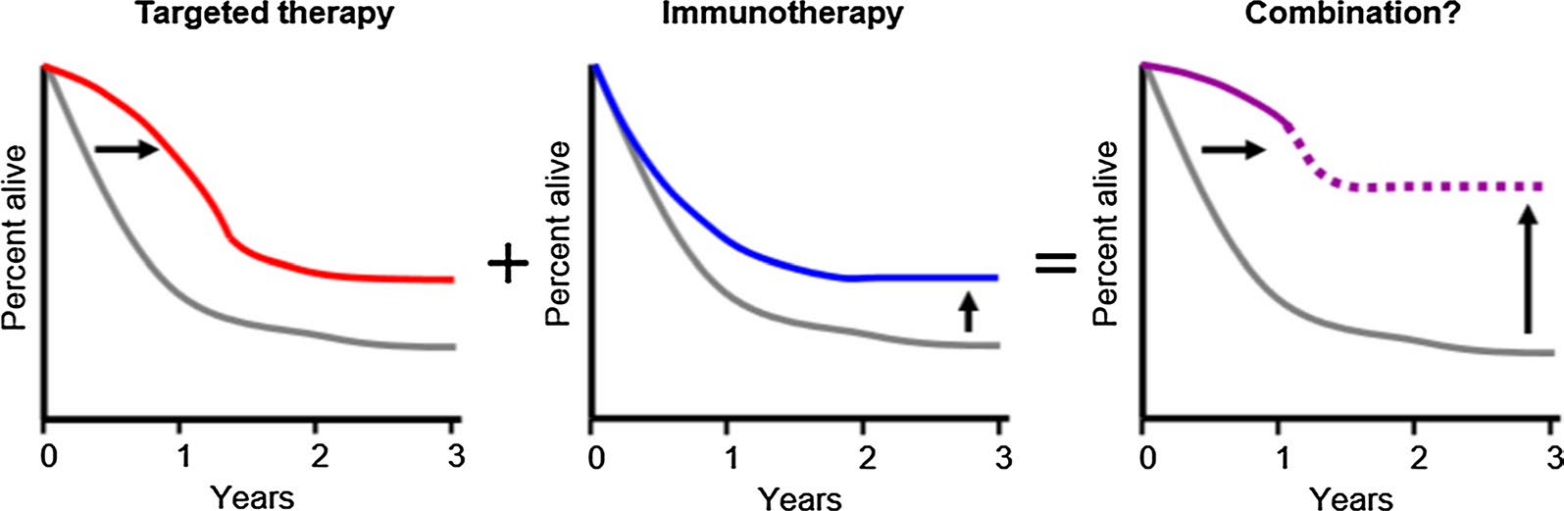
Melanoma survival curves depending on the type of therapy (modified from [150])

Thus, combining targeted therapy with immunotherapy may lead to enhanced anti-tumor response and result in durable responses and prolonged survival (Fig. 1).

The critical issue for designing effective combination of anti-BRAF and immunotherapy relies on determination of optimal selection of targeted and immune therapy agents and identification of biomarkers predictive of treatment response that will lead to rational combinatorial “regimens”. Retrospective analysis of overall survival (OS) revealed improvement in patients treated with ipilimumab compared with those treated with BRAF inhibitors first [64]. Another retrospective analysis from four major academic centers suggests that either BRAFi or anti-PD-1 may be effective regardless of treatment sequence in patients with BRAFV600-mutant melanoma, but clinical outcomes to front-line therapy are superior [65].

The data suggests that BRAFi as a salvage strategy may not be highly active in the subgroup that fails anti-PD-1 and front-line BRAF/MEK inhibitor therapy should be considered. Patients who benefited from BRAF-directed therapy for ≥ 6 months had a 34% overall response rate (ORR) to subsequent anti-PD-1 (11 of 32 patients responded). Patients who benefited for < 6 months subsequently had a 15% ORR to anti-PD-1 (4 of 26; p = 0.04). Shared “phenotype “to predict response for BRAFi and anti-PD-1 therapy may exist and studies of molecular characterization of “responders vs. resistant phenotype” are on the way. If successful, the identified profile may serve as a selection marker for prospective studies. It is suggested that there is a difference in the molecular profile of tumors responding to CTLA-4 vs PD-1. In the ideal scenario, molecular profiles identifying targeted therapy responders should not overlap with immune therapy responders to provide a benefit of combination therapy.

Resistance is a clinical problem and genetic mechanisms of acquired resistance are diverse [66]. Multiple genetic ways of reactivating the MAPK pathway have been identified. Besides, mechanisms of intrinsic resistance are not less diverse; one of the major drivers of intrinsic resistance seen in AXL-high at baseline [67].

Reduction in circulating tumor DNA (ctDNA) BRAF level is associated with tumor regression by RECIST: maximum ctDNA BRAFV600 level reduction occurs in Cycle 2 and 3 time points in vemurafenib treated patients and in at least Cycle 4 in patients treated with the combination of dabrafenib and trametinib. Average, a detectable increase in BRAFV600E level was seen ~ 50 days in patients treated with BRAF directed therapy prior to radiographic PD [68].

Recommendations from the “SWITCH 2.0” Trial for optimal strategies for treatment of melanoma patients include:

1. BRAFV600 positive tumor melanoma patients, frontline treatment.
2. Group 1 treated with BRAFi/MEKi until RECIST 1.1 progressive disease (PD) and then switched to ipilimumab/nivolumab, possibility to switch again to alternative BRAFi/MEKi.
3. Group 2 treated with BRAFi/MEKi until BRAF level first increase (or set at four cycles) and switched prior to RECIST 1.1 to ipilimumab/nivolumab with possibility to switch back to BRAFi/MEKi again if PD (alternative type of inhibitors).
4. Primary endpoint: PFS to ipilimumab/nivolumab between Groups 1 and 2, ORR, PFS from switch 1 to the end of second round of BRAF/MEK (where the strategy used).
5. Compare molecular profiles at diagnosis, after each line of therapy and at the time of ultimate resistance development (will differ based on intrinsic patient characteristics and types of therapy).

Building rational combinatorial “regimens” is the important factor. BRAFi effects on tumor microenvironment predict optimal combination with anti-PD-1/PD-L1 inhibition [69]. Preclinical data predicted synergy between MAPK targeting and PD-1/PDL1 inhibition [70, 71].

A phase Ib dose-escalation and -expansion study (NCT01988896) study combining atezolizumab and cobimetinib in metastatic melanoma suggested higher ORR/disease control rate [durable response rate (DCR), overall response rate (ORR) + stable disease] and longer PFS with the combination. Updated safety and efficacy data (Oct 12, 2016) showed that RECIST v1.1-confirmed ORR was 45.0% in patients with non-ocular melanoma (median duration of response was not reached); DCR was 75.0%; mPFS was 12.0 months (95% CI 2.8–not evaluable). ORR was similar for patients with BRAF-mutant and wild-type melanoma; adverse events (AEs) were experienced in all patients and related grade (G) 3–4 in 54.5% were most common; related serious AEs in 13.6% [72]. A phase 3 trial evaluating atezolizumab + cobimetinib vs anti-PD-1 therapy in patients with BRAF wild-type advanced melanoma is planned.

Targeted therapy with MEK inhibitor cobimetinib (cobi) + BRAF inhibitor vemurafenib in BRAFV600-mutant melanoma can result in anti-cancer immune activation and rapid clinical response. Inhibition of PD-L1 using atezolizumab can lead to anti-cancer immune activity and durable responses. Combining these agents may enhance antitumor immune activity and potentially improve both rate of clinical response and durability [69]. In this study 13/14 patients (93%) showed responses (RECIST v1.1), including 1 CR and 12 PRs and 11/13 pts continue in response. One patients with PR had a 100% reduction in target lesions. Responses were unconfirmed, and median DOR and PFS were not evaluable due to limited follow-up at the time of data cut (Feb 15, 2016). Functional biomarkers data of T-cell activation are ongoing.

Triple combination treatment was generally well tolerated as no unexpected AEs including grade 5 AEs occurred. All AEs were manageable and reversed with dose interruption and/or reduction. Treatment-related serious AEs including grade 3 blood creatinine phosphokinase levels increased (cobi-related), grade 4 sepsis (cobi-and/or vem-related), grade 3 diarrhea and ALT/AST levels increased (atezo- and/or cobi- and/or vem-related); all 3 patients continued on study treatments after interruption [73].

In conclusion, atezolizumab + cobimetinib demonstrated encouraging antitumor activity. However, it is not known whether the effect is additive or synergistic. Overall, ORR was 45% and mPFS was 12.0 months (atezolizumab monotherapy in cutaneous melanoma: ORR, 33%; mPFS, 5.5 months). The clinical benefit of atezolizumab + cobimetinib was seen regardless of BRAF status. BRAF mutant: ORR was 40%; mPFS, 11.9 months; BRAF wild type: ORR was 50%; mPFS, 15.7 months. Atezolizumab + cobimetinib had a manageable safety profile in metastatic melanoma, similar to that observed with atezolizumab alone or cobimetinib + vemurafenib combination. The combination atezolizumab + cobimetinib + vemurafenib had a manageable safety profile in patients with BRAF V600 mutant metastatic melanoma, but liver toxicity seems to be higher. The triple combination demonstrated promising antitumor activity, unconfirmed response rate was 83% (95% CI 64.2, 94.2).

The question remains whether to start the treatment with the combination of different agents including targeted agents, immunotherapy with checkpoints inhibitors, novel approaches) or add/switch on progression.

Other challenges in the optimal use of BRAF/MEK and immunotherapy are:

1. Incorporating novel agents-both targeted (AXLi) and immunotherapy (TLR9, T-VEC).
2. Postponing large phase 3 trials until we have identified better markers for patients’ selection.
3. Use of the data from early phase trials.
4. Need for model systems-surrogate markers (in vivo imaging, liquid biopsies).

### Innovative combination strategies: oncolytic and systemic therapy

Oncolytic therapy is a therapeutic modality of direct injection at the tumor site agents that may induce a local and systemic effect that is immunologically mediated and produce regression. The field of oncolytic virotherapy encompasses the use of viruses with natural or engineered tumor-selective replication to infect and kill tumor cells. The main intralesional (IL) agents currently in phase 3 trials are viral based (Talimogene laherparepvec, TVEC, HF-10, CAVATAK) and non-viral based (PV-10, IL-12). The mechanism of action is cell lysis (viral replication, chemical and mechanical ablation) and indirect “bystander response” by induction of innate immune response and adaptive immune response.

For an anticancer immune response to lead to effective killing of cancer cells, a series of stepwise events must be initiated and allowed to proceed and expand iteratively. We refer to these steps as the cancer-immunity cycle which manages the delicate balance between the recognition of nonself and the prevention of autoimmunity [74].

Intralesional oncolytic therapy in soft tissue and skin metastases has shown good safety profile and a durable response rate. Local–regional control of tumor growth is clinically important, whilst the systemic therapy may not always be possible or appropriate. Newer IL agents that have the ability to trigger a systemic immune effect can be clinically useful for combination treatment. Melanoma intra-lymphatic metastasis occurs in 3–10% of primary melanoma patients and it manifests clinically as local/in-transit recurrences. High risk groups for this presentation are characterized by thick, ulcerated, positive sentinel lymph nodes (SLN), lower extremity and greater than 50% risk of distant disease and death. Intralesional approaches may be applicable in melanoma treatment of these population of patients.

Current clinical trials with IL agents include monotherapy with PV-10 (phase III trial is ongoing, IL-12 administered by electroporation (EP), oncolytic Picornavirus, and Coxsackievirus A21 (CAVATAK™). Combination trials with TVEC, PV-10, HF10-oncolytic HSV1 and Reovirus (HF-10) are also ongoing.

PV-10 is an investigational new drug containing a proprietary injectable formulation of Rose Bengal disodium (10% RB). It is a small molecule fluorescein derivative lysing primary tumors by entering lysosomes, activating tumor-infiltrating lymphocytes at the local site and regression of distant tumors. Necrotic tumor cells have been shown to facilitate antigen presentation and the secondary tumors are rejected in immuno-competent animals. Responses are tumor specific and no immune response was reported in immuno-compromised animals. Adoptive transfer of spleen cells can convey immunity because T cell subsets have increased expression of IFNγ. A phase 2 study of intralesional PV-10 in refractory metastatic melanoma assessing efficacy and safety of PV-10 in 80 patients with refractory cutaneous or subcutaneous metastatic melanoma showed encouraging results. The best overall response rate for targeted lesion was 51%, and the complete response rate was 26%; median time to response was 1.9 months, and median duration of response was 4.0 months, with 8% of patients having no evidence of disease after 52 weeks [75]. Regression of bystander lesions strongly correlated with response in target lesions and responses occur early as 56% of lesions achieved complete response (CR) after 1–2 injections. An open-label, randomized controlled trial of single-agent intralesional PV-10 versus systemic chemotherapy or intralesional oncolytic viral therapy is currently ongoing to assess treatment of locally advanced cutaneous melanoma in patients who have failed or are not otherwise candidates for targeted therapy and have failed or are note candidates for at least one immune checkpoint inhibitor. An international multicenter, open-label, sequential phase study of intralesional PV-10 in combination with pembrolizumab in stage IV metastatic melanoma patients with at least one injectable cutaneous or subcutaneous lesion (NCT02557321) is ongoing. In the phase 1b portion of the study, all participants will receive the combination of IL PV-10 and pembrolizumab (i.e., PV-10 + standard of care). In the subsequent phase 2 portion of the study participants will be randomized 1:1 to receive either the combination of IL PV-10 and pembrolizumab or pembrolizumab alone (i.e., PV-10 + standard of care vs. standard of care).

The Intratumoral DNA-encoded IL-12 Electroporation (IT-pIL12-EP) is a DNA plasmid encoding interleukin-12 (IL-12), a potent pro-inflammatory cytokine. It is delivered directly to tumor in vivo by electroporation. Transfection of the plasmid stimulates local immune response and subsequently, systemic effect. Thirty patients with stage IIIB-IV melanoma received up to four cycles of IL-12 EP into superficial cutaneous, subcutaneous, and nodal lesions on days 1, 5 and 8 of each 12-week cycle. The treatment induced objective tumor responses in a significant proportion of patients (31%) and treatment was well tolerated [76].

CAVATAK, is a bioselected oncolytic strain of Coxsackievirus A21 (CVA21) is another oncolytic immunotherapy agent. Following intratumoral injection, CVA21 preferentially infects ICAM-1 expressing tumor cells, resulting in viral replication, cell lysis, and a systemic anti-tumor immune response. The phase II CALM study investigated the efficacy and safety of IT CVA21 in patients with advanced melanoma. The primary endpoint of the study was achieved in 38.6% evaluable patients with durable responses observed in both injected and uninjected melanoma metastases, suggesting the generation of systemic host anti-tumor responses [77].

Talimogene laherparepvec (T-VEC) is an intralesional oncolytic virus based on a modified herpes simplex virus type-1. It selectively targets tumor cells, causing regression in injected lesions and inducing immunologic responses that mediate regression at uninjected/distant sites. In a randomized phase 3 trial, T-VEC met its primary endpoint of improving the durable response rate vs granulocyte–macrophage colony-stimulating factor (GM-CSF) in patients with unresectable melanoma [78]. Responses were observed in injected and uninjected regional and visceral lesions. Exploratory analyses suggested survival differences in favor of T-VEC in patients with untreated or stage IIIB/IIIC/IVM1a disease. T-VEC therapy was generally well tolerated, the most common adverse events being flu-like symptoms [79].

OPTiM was a randomized, phase 3 trial of T-VEC or GM-CSF in patients with unresected melanoma with regional or distant metastases. The primary endpoint was durable response rate (DRR): partial or complete response (CR) continuously for ≥ 6 months starting within 12 months. Objective response rate with T-VEC was 26% (95% CI 21, 32%) with 11% CR, and with GM-CSF was 6% (95% CI 2, 10%) with 1% CR. DRR for T-VEC was 16% (95% CI 12, 21%) and 2% for GM-CSF (95% CI 0, 5%), p < 0.0001. DRR by stage (T-VEC, GM-CSF) was IIIB/C (33, 0%), M1a (16, 2%), M1b (3, 4%), and M1c (8, 3%). Interim OS showed a trend in favor of T-VEC; HR 0.79 (95% CI 0.61, 1.02) which did not reach statistical significance. Most common adverse events (AEs) with T-VEC were fatigue, chills, and pyrexia. Serious adverse events (AEs) occurred in 26% of T-VEC and 13% of GM-CSF pts. No ≥ grade 3 AE occurred in ≥ 3% of pts in either arm [80].

Preliminary data suggest higher CR and OR rates than either agent alone and earlier responses after ipilimumab initiation during T-VEC + ipilimumab than with ipilimumab alone [81]. A phase 1b demonstrated that of 17 pts with investigator assessed response, ORR was 41% (24% CR, 18% PR); 35% had stable disease (SD). Median time to response was 2.9 months. Activated CD8 T cells numbers significantly increased from baseline 1.8× after T-VEC alone and 2.9× during T-VEC + ipilimumab treatment; Gr 3/4 AEs occurred in 32% as determined by flow cytometry [82].

ORR was higher for T-VEC in combination with ipilimumab vs ipilimumab alone [83]. Confirmed ORR was 35.7% (T + I) and 17.5% (I); unconfirmed ORR was 50% (T + I) and 27.5%. AEs were comparable between arms except for increased fatigue, chills, and pyrexia in the T + I arm [83].

The combination of T-VEC and pembrolizumab demonstrated improved clinical benefit in a phase 1b/2 study in unresectable stage IIIB-IV melanoma with all patients having started on T-VEC + pembrolizumab ≥ 6 months prior [84]. Per immune related criteria (irRC), in 21 pts, confirmed/not yet confirmed objective response rate (ORR) was 48%/57%; CR rate was 14%/24%. Median time to response was 17 wks. Circulating CD8+ T cells including those expressing defined immune modulatory receptors (e.g., Tim3, BTLA) became elevated during therapy with T-VEC initially but decreased after pembrolizumab began on d 36 [84].

A phase 1b on subjects treated with MK-3475 (pembrolizumab) until CR disease progression per irRC, or intolerance of study treatment, up to a maximum of 24 months of study treatment is ongoing (NCT02263508). In phase 3, subjects will be treated with T-VEC plus pembrolizumab (arm 1) or placebo plus pembrolizumab (arm 2) until 24 months from the date of the first dose of pembrolizumab or end of treatment due to disappearance of injectable lesions, complete response, disease progression per irRC-RECIST or intolerance of study treatment.

HF10 is not genetically engineered but is a nonselective clone from the non-neuroinvasive HSV-1 strain HF. Apart from loss of UL56 gene, sequencing of HF10 revealed an overall 99.1% similarity to HSV-1 strain 17, with mutations in genes involved in regulation of syncytia formation including UL1, UL20, UL22, UL24, UL27, and UL53. HSV strains can spread from cell-to-cell via infection across the junctions between the membranes of adjacent cells (wild-type strains (syn+) or by fusion of the infected cell with adjacent uninfected cells leading to the formation of multinucleated polykaryocytes or syncytia [syncytial mutant strains (syn)]. Interestingly, HF10 induces syncytia formation in vitro, and did not result in the cytopathic effect observed in hrR3 infected cells [85]. It is characterized by greater replication ability, resulting in lower effective dose and no toxicity resulting from inserted exogenous gene (ex. GM-CSF). Attenuation of neurovirulence to be attributable to the lack of the UL56 gene (lack of UL56 gene decreases HSV-1 pathogenicity without affecting viral replication ability). In addition to local oncolytic tumor destruction, systemic anti-tumor immune response observed.

Preliminary data of the antitumor activity of HF10 + ipilimumab combination in an ongoing phase 2 trial in melanoma on 43 patients enrolled and treated at abstract data cut-off 01 Feb16 show that majority of HF10-related AEs are ≤ G2, similarly to HF10 monotherapy and consistent with other oncolytic viruses. No DLTs or ≥ G4 AEs were reported. G3 AEs were experienced by 11.6% of pts. Of 37 efficacy evaluable patients, BORR by irRC at 24 weeks is 37.8% (13.5% CR and 24.3% PR) and disease stability rate is 56.8% (18.9% SD) [86].

Pro and con arguments of the role for IL monotherapy are reported in Table 2.

**Table 2:** Potential role of intralesional monotherapy

Yes	No
Not all patients are candidate for systemic therapy (co-morbidities, toxicity)	Systemic therapies in 2015 are safe and effective
After progression on other therapies	Melanoma is a systemic disease
Alternative to surgery?	Surgery is an instant CR
Neoadjuvant potential	Not yet proven

In conclusion, soft tissue and cutaneous metastases are a major clinical problem in melanoma. Oncolytic intralesional approaches may have value as local direct effect, systemic immune effect and low toxicity. Several agents in development appear promising such as TVEC that was approved by US and EU regulators. Combination therapies are likely to be the future and may be the best way to integrate them into clinical practice.

## System biology session: immunology

### Rational design of combination immunotherapy

Effective immunotherapy is a balance between induction of immune response (NK, B cell, CD4 cells, CTL) and inhibition of suppression (Tregs, MDSC, TAM, IDO). Biologically rational design of immunotherapeutic combination is crucial for the success of such approach.

The PD-1 receptor is a negative regulator of T-cell effector mechanisms that limits immune responses against cancer: lambrolizumab was tested in patients with advanced melanoma and showed a high rate of sustained tumor regression, with mainly grade 1 or 2 toxic effects [87]. In patients with melanoma, ipilimumab prolongs overall survival, and nivolumab produced durable tumor regression in a phase 1 trial. Based on their distinct immunologic mechanisms of action and supportive preclinical data, a phase 1 trial of nivolumab combined with ipilimumab in patients with advanced melanoma showed a rapid and deep tumor regression in a substantial proportion of patients; a total of 16 out of 53 patients had tumor reduction of 80% or more at 12 weeks, including 5 with a complete response [88]. Accordingly, preliminary data of early clinical trials of such combination proved better than the effect of each of the single agents.

Other immunomodulatory agents that may come into play in the spectrum of combination therapy include immune agonists antibodies of activating receptors such as anti-OX40 and anti-GITR. Anti-OX40 enhances T cell responses and is associated with increased T cell expansion/proliferation, effector function, T cell survival, T cell memory development as well as enhancing vaccine therapeutic effect. Accordingly, the combination of anti-OX40 and anti-PD-1 is a rational strategy in immune therapy combination development. Few clinical trials are currently being conducted combining these two agents. Interestingly, we found that the sequence of such combination is crucial for its success or failure. In addition, combining OX40 agonist antibody enhances specific immune response in pre-primed animals with tumor specific antigens. This enhancement leads to better tumor response that is immune-dependent. However, when anti-PD-1 is combined concomitantly with agonist anti-OX40, surprisingly, we found that it completely abrogates the immune response of OX40 and hence eliminates the tumor response induced by the agonist antibody [89]. We also found, that blocking PD-1/PD-L1 pathway while activating OX40 drives the CD8 T cells into apoptotic cell death [89]. Accordingly, it is crucial to know that combining two immune modularity agents is not a straightforward approach and such combination may not induce a desirable response if not treated in the proper sequence. In a combination approach, any of the agents could lead to changes within the tumor microenvironment that produce a specific immune effect which may influence the outcome of the second immunomodulator in a potentially positive or a negative manner.

Naturally occurring T regulatory cells (Tregs) express the transcription factor Foxp3, which is a master regulator of Tregs development and function. The Foxp3 gene was first identified as the defective gene in the mouse strain Scurfy. Scurfy is an X-linked recessive mutant that is lethal in hemizygous males within a month after birth. Hypoxia, or low oxygen tension, is a major regulator of tumor development and aggressiveness. Accordingly, strategies to decrease Tregs are important to be included within the combination repertoire to further enhance the outcome of therapeutic approaches. Cyclophosphamide used in low metronomic doses have been shown to decrease Tregs in the microenvironment and enhance the anti-tumor immune effect [90]. Based on that, we found that combining antigen-vaccine with anti-PD-1 and a single dose of low-dose cyclophosphamide can improve immune outcome [91]. Utilizing Listeria as a platform for cancer vaccine to generate antigen-specific anti-tumor immune response, we found that Listeria exhibit a bystander immune response leading to the decrease in the ratio of Tregs to CD4 T-cells [92]. It has been reported that anti-glucocorticoid-induced tumor necrosis factor receptor (GITR) agonist antibodies leads to tumor regression only in the context of Tregs depletions [93]. Based on that and since Listeria-based immunotherapy is able to decrease the ratio of Tregs to CD4 T-cells, we hypothesized that combining Listeria-based vaccine with GITR-agonist antibody would exhibit synergistic anti-tumor immune response. Indeed, listeria-based (Lm) immunotherapy combined with agonist anti-GITR antibody provide a potent synergistic treatment strategy that simultaneously targets both the effector and suppressor arms of the immune system, leading to significantly improved anti-tumor efficacy [94].

In summary, unlike most of chemotherapeutic approaches to cancer, combination immunotherapy is not a random approach of drug coupling, but rather it should be based on biologically based rational approach to therapy that is intended to redesign the immune composition of the tumor microenvironment.

### Bioinformatics approaches to investigate mechanisms driving the non-T cell-inflamed tumor microenvironment

Tumors with substantial susceptibility to anti-PD-1 antibody are numerous (melanoma, non-small cell lung cancer, renal cell carcinoma—clear cell carcinoma etc.) and registrational trials are on-going in many cancer histologies (gastroesophageal cancer, hepatocellular carcinoma, mesothelioma etc.) [95].

The T cell-inflamed tumor microenvironment is characterized by expression of immune-inhibitory pathways and predicts patient outcomes to immunotherapy [96, 97]. The molecular mechanisms that explain the T cell-inflamed versus non-inflamed tumor microenvironments could include though not necessarily be limited to: (1) somatic differences at the level of tumor cells characterized by distinct oncogene pathways activated in different patients and mutational landscape and antigenic repertoire; (2) germline genetic differences at the level of the host (patient) characterized by polymorphisms in immune regulatory genes; (3) environmental differences characterized by commensal microbiota. Data for each of these is now available for melanoma and investigations are on-going to spread these concepts across tumor types.

Regarding cancer cell intrinsic properties and specifically distinct oncogene pathways, some of T cell exclusion have been elucidated. Only a subset of patients responds to blockade of immune-inhibitory receptors treatments and the therapeutic benefit is preferentially achieved in patients with a pre-existing T-cell response against their tumor, as evidenced by a baseline CD8+ T-cell infiltration within the tumor microenvironment. It is critical to understand molecular mechanisms of a spontaneous anti-tumor T-cell response in melanoma-cell-intrinsic oncogenic pathway that contributes to a lack of T-cell infiltration in melanoma [98]. The correlation between activation of the WNT/β-catenin signaling pathway and absence of a T-cell gene expression signature was the first tumor-intrinsic molecular signaling pathway identified to mediate immune exclusion. Activated β-catenin signaling results in T-cell exclusion and resistance to anti-PD-L1/anti-CTLA-4 monoclonal antibody therapy [98]. On a mechanistic level, β-catenin represses CCL4 chemokine, leading to lack of Batf3+ DC recruitment, failed T cell priming and response to checkpoint blockade [98]. Genetic landscape of the T cell-inflamed tumor microenvironment across The Cancer Genome Atlas (TCGA) solid tumors was investigated in order to describe the spectrum of the T cell-inflamed tumor phenotype across histologies [99]. Studies of mutational burden compared with the T cell-inflamed tumor microenvironment showed no correlation between gene expression and mutational burden in any cancer type [99]. Antigen is not rate-limiting in non-T cell-inflamed tumors that lack of spontaneous immune infiltration in solid tumors is unlikely due to lack of antigens. Rather a clear linear association with antigen presenting machinery and Batf3 lineage DCs are suggested. As an example of this approach, gene expression in testicular germ cell tumor (GCT) revealed a T-cell-inflamed tumor microenvironment in 47% of testicular GCTs, including seminoma (83%) and nonseminoma (17%) tumor subtypes [100]. Expression of alpha-fetoprotein (AFP) RNA correlated with lack of the T-cell signature, with increasing AFP RNA inversely correlating with the inflamed signature and expression of IFNγ-associated genes. These data suggest that GCTs can respond to anti-PD-1 and that gene expression profiling supports investigation of immunotherapy for treatment of GCTs [100]. The presence of intratumoral CD8+ T cells has been associated with clinical benefit to immunotherapy and patients can be categorized based on the presence or absence of a T cell-inflamed tumor microenvironment. Molecular mechanisms underpinning the absence of a T cell response are beginning to be understood with identification of the WNT/β-catenin pathway playing a major role in melanoma including activating mutations CTNNB1 and Inactivating mutations in negative regulators of Wnt pathway (Axin1, Axin2, APC1, APC2) [101]. Pathway activation without mutation includes overexpression of Wnt ligands, Fzd receptors, β-catenin [101]. Other tumor-oncogenic pathways correlating with T-cell exclusion in the non-T-cell-inflamed tumor microenvironment and the resistance to immunotherapies are also under investigation. Upregulation of genes encoding immune checkpoint proteins PD-L1, IDO, FOXP3, TIM3, and LAG3 was associated with T-cell-inflamed tumors, suggesting potential for sensitivity to checkpoint blockade. Conversely, β-catenin, PPAR-γ, and FGFR3 pathways were activated in non-T-cell-inflamed tumors [102]. Furthermore, PTEN loss in melanoma associates with non-T cell-inflamed tumor microenvironment and resistance to anti-PD-1 [103].

The next hurdle in cancer immunotherapy is overcoming the non-T cell-inflamed tumor microenvironment. Therefore, a number of clinical trials are ongoing on different targets, promoting innate immunity/type I IFNs like intratumoral STING agonists, inducing tertiary lymphoid structures and modulate stroma (anti-CD40). Examples are phase 1 study of stereotactic body radiotherapy followed by pembrolizumab in advanced solid tumor, phase 1 study of SEA-CD40 in advanced malignancies, phase 1b/2 study of BBI608 in combination with immune checkpoint inhibitors in advanced solid tumors and phase II dual cohort study of gut microbiota modulation with pembrolizumab in melanoma.

In conclusion, anti-PD-1 or PD-L1 immunotherapy is becoming standard therapy in many tumor types. T cell gene signatures correlate well with available biomarkers (including gene expression, mutational load, PD-L1, TCR clonality) and clinical benefit. Future immunotherapy drug development should be stratified toward engaging either the inflamed or non-inflamed tumor microenvironment (inflamed like IDO, Tregs, MDSCs and non-inflamed like RT, STING, CD40) and molecular pathways leading to immune exclusion (e.g., β-catenin/PTEN).

### The gut microbiome and cancer therapeutics

The gut microbiota is composed mostly of bacteria, but also fungi, archaea and viruses. Evolutionary development of the host immune system has been closely associated with the microbiota in a symbiotic relationship (eubiosis). This diverse microbiome is associated with many diseases including asthma, allergy and inflammatory bowel disease, and is involved in numerous functions including, digestion of complex carbohydrates, direct competition for limited nutrients and occupation of ecological niches that may otherwise be colonized by pathogenic microorganisms [104]. Furthermore, the gut microbiota can affect distant organs [105]. Dissecting the relationship between the microbiota, pathogens and the host may provide novel insights into disease pathogenesis, as well as novel avenues for the prevention and treatment of intestinal and systemic disorders including cancer.

During cancer treatment, there is a complex interplay between the gut microbiota and anticancer therapies. These therapies can exert cytotoxic effects on intestinal bacteria, leading to dysbiosis. However, the gut microbiota can in turn influence both the therapeutic activity and the side effects of anticancer agents, via pharmacodynamics and immunological mechanisms [106]. The antitumor efficacy of immune checkpoint inhibitors including anti-CTLA-4 has been demonstrated to require gut microbiota, in particular, gram-negative bacteria [107]. *Burkholderia cepacia* acts in conjunction with *Bacteroides fragilis* to restore sensitivity to anti-CTLA-4. Adoptive T cell transfer of T cells primed with *B. fragilis* ameliorates the antitumor effects of CTLA-4 blockade in germ free mice. Anti-CTLA-4 compromises the homeostatic equilibrium between Intestinal Epithelial Cells (IEC) and intraepithelial lymphocyte, leading to the apoptotic demise of IEC in the presence of microbial products. Compensation of mice with *B. fragilis* + *B. cepacia* was able to protect against subclinical toxicity. Furthermore, we saw an increase in IFNγ and a decrease in IL-10 production in *B. fragilis*/Bacteroides thetaiotaomicron-specific memory CD4+ T cell responses in metastatic melanoma patients post-CTLA-4 blockade. Feces from metastatic melanoma patients were analysed and grouped into three clusters (A, B and C) based on genus composition. Germ free (GF) mice transplanted with feces from Cluster C patients had a significantly greater response to CTLA-4 blockade compared to mice which received Cluster B feces and were found to facilitate the outgrowth of beneficial *B. fragilis*. The efficacy of anti-CTLA-4 therapy in Cluster B transplanted mice could be improved by compensation mice with certain bacteria. In conclusion, gut microbiota impacts therapy-induced antitumor immunosurveillance and that the therapeutic coverage of immune checkpoint blockade could be broadened when a favorable microbiota is present.

### Next target for immune checkpoint blockade

There is ample evidence that high-level spontaneous and vaccine-induced tumor antigen-specific T cells may exist in patients with advanced and progressive melanoma. This paradoxical coexistence of T cell immune responses with melanoma progression has led us to investigate the multiple immunoregulatory pathways driving T-cell dysfunction in the tumor micro environment (TME). The upregulation of inhibitory receptors by T cells chronically activated by tumor cells in the TME represents a major mechanism of tumor-induced T cell dysfunction. Targeting inhibitory pathways with blocking antibodies have transformed the standard of care for patients with melanoma and other solid tumors. Anti-PD-1 antibodies are a potent therapy for melanoma, which provide clinical benefits to 30–40% of patients with advanced melanoma. Beyond PD-1, group at the University of Pittsburgh has worked on identifying additional inhibitory pathways that may cooperate with PD-1 to dampen T cell responses to melanoma. There are numerous inhibitory receptors expressed by T cells in the TME that bind to their respective ligands expressed by antigen-presenting cells and tumor cells [108].

The rationale for optimal combinatorial immune checkpoint blockades is based on the identification of inhibitory or activating receptors expressed by a significant number of tumor antigen-specific CD8+ T cells. The evidence exists of additive/synergistic effects on tumor-antigen specific CD8+ T cell expansion and function upon dual blockade targeting non-redundant inhibitory pathway. Mouse tumor models support the in vivo efficacy of the dual blockade with a nontoxic and safe profile. Based on this hypothesis, the group has focused on the novel T cell inhibitory receptor with immunoglobulin (Ig) and immunoreceptor tyrosine-based inhibition motif (ITIM) domains called TIGIT. TIGIT is expressed by T cells and NK cells. It competes with the costimulatory molecules CD226/DNMA-1 for binding to the same ligands CD155/PVR and CD112 expressed by antigen-presenting cells and tumor cells [109].

TIGIT was shown to be co-expressed with PD-1 by tumor antigen-specific CD8+ T cells in melanoma and TIGIT ligands are highly expressed in metastatic melanoma [110]. Dual TIGIT/PD-1 blockade increases the expansion and function of human tumor antigen (TA)-specific CD8+ T cells. In addition, others have also observed that dual PD-1/TIGIT blockade promotes tumor regression in multiple mouse tumor models [110].

TIGIT is also upregulated by human Tregs in the TME, and TIGIT high CD4+ TILs are highly immunosuppressive. Targeting TIGIT with Fc-engineered mAbs may prove efficacious to deplete Tregs in patients with advanced melanoma.

In conclusion, TA-specific CD8+ T cells in the periphery and at the tumor sites co-express a number of inhibitory receptors in addition to PD-1, including TIGIT. TIGIT ligands are highly expressed in the TME of melanoma and many other solid tumors. Dual PD-1/TIGIT blockade augments the expansion and function of human TA-specific CD8+ T cells in vitro and promotes tumor regression in multiple mouse tumor models. These data served as the rationale for ongoing clinical trials with dual PD-1/TIGIT blockade in patients with advanced cancers, including melanoma.

### Advancing dendritic cell cancer immunotherapy

In stage D0 prostate cancer, PSA recurrence without evidence of measurable disease is a clinical problem with limited treatment options. Immune therapy could delay the initiation of androgen deprivation therapy. In fact, a pilot study of vaccination with epitope-enhanced T cell receptor alternate reading frame protein (TARP) and TARP peptide-pulsed dendritic cells for the treatment of stage D0 prostate cancer has recently been completed [111]. TARP is a novel 58 amino acid protein, expressed on 85–95% of prostate cancer specimens, which originates from prostate epithelial cells, not infiltrating T cells. It is expressed on normal prostate epithelium and over-expressed in prostate cancer. TARP expression is associated with conventional markers of unfavorable and more aggressive tumor behavior. The study investigated TARP peptide vaccination’s impact on the rise in PSA [expressed as slope log (PSA) or PSA doubling time (PSADT)], validated tumor growth measures, and tumor growth rate in men with Stage D0 prostate cancer. HLA-A*0201 positive men were randomized to receive epitope-enhanced (29-37-9V) and wild-type (27–35) TARP peptides administered as a Montanide/GM-CSF peptide emulsion or as an autologous peptide-pulsed dendritic cell vaccine, every 3 weeks for a total of five vaccinations with an optional 6th dose of vaccine at 36 weeks based on immune response or PSADT criteria with a booster dose of vaccine for all patients at 48 and 96 weeks [111]. Per protocol, Peripheral Blood Mononuclear Cells (PBMCs) were collected by apheresis, PBMCs were enriched for monocytes by counter-flow elutriation (20–80%) and aliquots of the monocytes were cryopreserved. Subsequently, thawed monocytes culture for 3 days with IL-4 + GM-CSF and then culture for 1 day with LPS + IFN-1. On the last day of culture the DCs were pulsed with HLA-A2-restricted TARP peptides, harvested and infused. 72% (n = 41) of patients reaching 24 weeks and 74% reaching 48 weeks had a decreased slope log (PSA) compared to their pre-vaccination baseline (p = 0.0012 and p = 0.0004 for comparison of overall changes in slope log (PSA), respectively). TARP vaccination also decreased by 50% in median tumor growth rate (g): pre-vaccine g = 0.0042/day, post-vaccine g = 0.0021/day (p = 0.003). 80% of subjects exhibited new vaccine-induced TARP-specific IFNγ ELISPOT responses but these responses did not correlate with decreases in slope log (PSA). Thus, vaccination with TARP peptides resulted in significant slowing in PSA velocity and reduction in tumor growth rate in most of patients with PSA biochemical recurrence [111].

This study suggests TRAP DC vaccines could be improved by understanding lot-to-lot variability and better understanding of the mechanisms of action and biomarkers associated or predictive of clinical response. TARP DCs are autologous therapies and a unique product is manufactured for each vaccine, consequently, patient factors can contribute to product variability. Sources of variability of cellular therapies are starting materials (patient biology in terms of genetic, gender, race and age), patient disease (type, stage and treatment); manufacture related (starting material, separation, response to stimuli and metabolism).

A very recent study a subset of stage D0 prostate cancer patients treated with TRAP DC vaccines aimed to identify DC markers correlating with clinical and immunologic response to the vaccination regimen. TRAP DCs from 18 vaccinated patients were extensively characterized [112]. Peptide-pulsed DC preparations were analyzed by gene expression profiling, cell surface marker expression and cytokine release secretion, and correlated with clinical and immunologic responses. Characteristics of the final DC product were: > 95% for all products (CD80, CD83, CD86, CD123, CD11c, CD38, CD54, and HLA-DR); variable expression (CD14: range 14–90%, CCR7: range 5–90%, viability: range 37–91%, DC yield range 6–48%). Comparison of TARP DCs associated with PSA clinical response and non-response showed in responders a trend toward higher levels of CCR7 and a trend toward lower levels of CD14. Hierarchical clustering and principal component analysis of the gene expression data revealed that DCs clustered by patient with no separation of responders and non-responders. A class comparison of clinical responders and non-responders found 55 differentially expressed genes (false discovery rate = 65%). The weighted gene coexpression network analysis (WGCNA) method was used for further analysis of the gene expression data. WGCNA is a systems biology method, describing correlation patterns across microarray samples, it is not biased and it finds clusters or modules of highly correlated genes. This analysis found that DCs showing lower expression of a tolerogenic gene signature induced strong antigen-specific immune response and slowing in PSA velocity, a surrogate for clinical response. These DCs were also characterized by lower surface expression of CD14, secretion of IL10 and MCP-1 (CCL2), and greater secretion of MDC (CCL22). When combined, these four factors discriminate DCs inducing strong immunologic response.

In conclusion, tolerogenic TARP DCs are associated with a poor immunological and clinical response and candidate DC potency markers include CD14, IL-10, CCL2 and CCL22. However, lot-to-lot variability is a critical issue for DC-based immunotherapies. There is no best method to identify cell therapy biomarkers and both hypothesis and discovery driven approaches are effective. Product variation can be used to better understand mechanism of action and identify potency markers. Work is ongoing to improve the consistency of DC-based immunotherapies.

## Biomarkers session

### The promising alliance of electrochemotherapy and immunotherapy

Electroporation (EP) consists in the delivery of a limited number of short and intense electric pulses which are defined by an intensity E and a duration t. Above a certain threshold of the E and/or t parameters, cell membrane defects appear and result in cell permeabilization. After a given lag time, cell membrane integrity is restored leading to cell survival.

Electrochemotherapy (ECT) consists in the delivery of short and intense electric pulses following the administration of non- or low-permeant cytotoxic drugs, such as bleomycin. Cell membrane permeabilization permits the drug to enter the target cells and eventually to trigger cell death through multiple DNA breaks, which are lethal for dividing cells.

Electroporation and electroporation-based therapies are promising approaches for the treatment of cancer. The following medical applications of electroporation have already been developed and brought to clinics:

1. Electroporation to transfer drugs and small molecules is the combination of EP and cytotoxic drugs that do not freely cross the plasma membrane. ECT was the first application that reached the clinical stage. ECT works very efficiently and without major side effects. It selectively kills the tumor cells and spares the normal non-dividing cells in the volume exposed to electric pulses (EPs) (usually eight short pulses of 100-μs duration). However, ECT remains a local treatment with no obvious effects on distant metastases.
  Two anti-cancer molecules bleomycin and cisplatin have met these prerequisites and are currently used in the clinical practice of ECT. These drugs, once internalized into cells via the local delivery of EPs, generate DNA lesions, either both single-strand and double-strand DNA breaks (if bleomycin is used) or adducts and intra-strand and inter-strand DNA bonds (if cisplatin is used), ultimately leading to cell death.
  After cell electroporation, large amounts of the cytotoxic molecules enter cells by diffusion, regardless of the cell type. Electroporation thus turns bleomycin and cisplatin into very efficient drugs in all tumor types, as verified in preclinical and clinical studies. A wide range of tumors has been treated by ECT, mainly using bleomycin. These now include primary tumors (basal cell carcinoma) and metastases of head and neck carcinomas, Kaposi’s sarcoma, breast adenocarcinoma, and melanoma. Clinical trials are nowadays addressing the treatment of deep-seated tumors (primary pancreatic carcinoma and bone, liver, and brain metastases).
  In addition, ECT demonstrates potent anti-vascular effects. A transient vasoconstriction is observed following EPs delivery alone, and moreover, the endothelial cells forming tumor blood vessels are also sensitive to ECT (as any proliferative cell in the treated region). Consequently, these phenomena result in tumor starvation (lack of oxygen and growth factors) and thus contribute to cancer cell death.
  Nowadays, ECT is used in routine in about 140 European cancer centers.
  Technology was developed in the Cliniporator EU project. Then, the EU-funded ESOPE clinical study demonstrated an objective response rate of 85% in ECT treated tumor nodules, regardless of the tumor histology and drug used or route of its administration [113]. It also established the standard operating procedures for ECT use in the clinic i.e., a series of eight electric pulses (EPs) of 100 μs and appropriate field amplitude must be delivered using either invasive (EPs of 1000 V/cm) or non-invasive electrodes (EPs of 1300 V/cm), depending on the depth and on the size of the nodules to treat. Recently, in addition to treating cutaneous and subcutaneous tumors the treatment of deep-seated tumors was applied in clinical trials. Clinical trials are also ongoing for bone metastases, liver metastases, brain tumors, pancreas, colorectal cancers (endoscopic electrodes), as demonstrated in number of published reports.
2. Electroporation to transfer nucleic acids inside the cells, the electroporation-based gene transfer, constitutes a subcategory of gene therapy. Electroporation mediated gene therapy, namely, electrogenetherapy (EGT), is an approach rapidly expanding in cancer and non-cancer therapeutic domains. Gene electrotransfer can be achieved by first permeabilizing the cell membrane thanks to short and intense electric pulse deliveries and second by driving electrophoretically the DNA toward the electroporated membrane thanks to one or several long and low-voltage electric pulses. It is expected that a protein of interest is produced and epitope presentation occurs on MHC molecules.
  DNA vaccination consists of the administration of a DNA encoding a cancer antigen of interest. Encoded antigen will be responsible for the generation of a pool of specific B and T cells, from which some will remain as memory cells for long-term protection. Tumor-specific CD8+ T cell generation, associated with the secretion of Th1 cytokines (e.g., tumor necrosis factor alpha, TNFα and IFNγ) was demonstrated. Gene electrotransfer is one of the most efficient non-viral techniques and has proven its efficiency in many tissues, should they be superficial (e.g., skin) or internal (e.g., liver and muscle). The main limit to the use of gene electrotransfer is related to its application to not easily accessible organs. The two most advanced EGT strategies are related to immunotherapy, namely, DNA vaccination and cytokine-based anti-cancer therapies. Intratumoral administration of an IL-12-encoding plasmid in conjunction with EPs demonstrated local and systemic anti-tumor effects and improved cure rates of tumors in animal models as well as in patients.
3. Irreversible electroporation (IRE) consists in the use of excessive electroporation to cause cell death. Different approaches can lead to this outcome including the use of very long or very intense electric pulses. This local ablative treatment is not selective against the tumor cells, killing also the normal cells in the volume of tissue exposed to the EP [114].
4. There is also an interest in the combination of anti-tumor ECT with immunotherapy for long-term and systemic anti-tumor responses. Intratumoral recruitment of dendritic cells (DCs) expressing CD80/CD86 maturation markers, circulating monocytes and splenic T lymphocytes was observed after ECT treatment in animal model. These studies highlight immune system activation after the treatment. Recently, the mechanisms underlying this immune activation were identified. ECT induces an immunogenic cancer cell death (ICD) through the liberation of ATP and HMGB1 and the translocation of calreticulin to the cell surface. Immunogenic cell death is responsible for the generation of tumor-specific T cells [113, 114], that are able to kill non-ECT-sensitive cancer cells within the primary tumor. Interestingly, cancer stem cells, thought to be responsible for cancer recurrence and metastasis, seem sensitive to both extracellular ATP and T cell recognition. These tumor-specific T cells can also target metastatic nodules, although there is a lack of direct evidence in the absence of a complementary immune stimulation. Immunotherapy agents (e.g., cytokines, therapeutic antibodies, immune checkpoint blockers, and genes) mount immune responses that are synergistic with the one triggered by ECT. Overall, this ECT-driven immune activation might be responsible for controlling local relapses and metastatic spread.
  Antigen presenting cells (APCs), mostly DCs, are of great importance for the outcome of vaccination as they ensure effective T cell priming and maintenance. Therefore, EPs appear to play a pivotal role in anti-cancer DNA vaccination, not only by enhancing the transgene expression but also by recruiting APCs in the electroporated tissues: in vivo electroporation can also serve as an adjuvant for DNA vaccination [115]. ECT is more efficient in immunocompetent mice as compared with immunodeficient animals [114, 116]. It has been applied in humans as monotherapy and in combination approaches [117–119]. Recently Mozzillo et al. reported that the combination of ipilimumab and ECT may be beneficial for the treatment of metastatic melanoma [118]. The study reports that the volume of distant non-ECT-treated tumors decreased or was stabilized in 9 patients out of 15, possibly through ipilimumab-induced Tregs depletion. Local ECT treatment of cutaneous lesions of melanoma was followed by ipilimumab administration resulting in the complete regression of all the cutaneous and visceral metastases for at least 1 year. Interestingly, vitiligo-like lesions developed exclusively around the sites of previous ECT, suggesting that a prior ECT-driven immune activation was enhanced by ipilimumab [120]. Electrochemotherapy in combination with ipilimumab or pembrolizumab or nivolumab was also studied [119]. Combination of ECT with immunotherapy is likely to transform the ECT from a local to systemic therapy [121]. ECT has numerous advantages such as: no side effects; is not immunosuppressive; stimulates the immune system; rarely causes bleeding even after needles insertion; possess anti-hemorrhagic effects; high specificity against tumor cells; treatment planning procedures are available; standardized equipment is available; has reasonable cost; is efficient and fast; and requires minimal post treatment care. In conclusion, ETC-based strategies represent highly promising approaches to induce anticancer systemic effects and benefit for patients.

### HLA class I antigen processing machinery defects in malignant cells in the era of immunotherapy of malignant diseases with immune check point inhibitors

The results of a growing number of clinical trials in large populations of patients with many types of malignancies have convincingly shown that immunotherapy with checkpoint inhibitors as monotherapy or in combination with other agents including immunomodulators can induce long lasting clinical responses in patients with malignant diseases. However, these therapeutic effects occur only in a small number of the treated patients with a given type of cancer. Furthermore, the frequency of responses is markedly different among the various types of cancer. Several lines of evidence including the increased lymphocyte infiltration in tumors, the correlation between mutation load in tumors and clinical outcome in patients treated with immune checkpoint inhibitors are consistent with the possibility that clinical responses reflect the recognition and elimination of cancer cells by cognate T cells unleashed by immune check point inhibitors. The interactions between tumor cells and cognate T cells are mediated by HLA class I-tumor antigen derived peptide complexes. Their synthesis and expression require a fully functional HLA class I antigen processing machinery (APM). Specifically, proteasome isoforms and their active subunits degrade down mostly, although not exclusively, endogenous proteins to peptides with the correct length and sequence for HLA class I binding. Proteasomal degradation products are shuffled by the heterodimeric transporter associated with antigen processing (TAP) complex into the lumen of the endoplasmic reticulum for loading of beta2 microglobulin (β2m)-associated HLA class I α and β chain dimers with the help of the chaperone molecules ERp57, calnexin, calreticulin and tapasin. The resulting trimers then travel to the plasma membrane and tumor antigen derived peptides are presented to cognate T cells.

The role played by HLA class I APM in the response to therapy with immune checkpoint inhibitors has rekindled interest in the characterization of the defects in HLA class I APM component expression and/or function in malignant cells, since these defects may represent one of the mechanisms underlying the resistance to immune checkpoint inhibitor-based therapy. Extensive review of the literature describing HLA class I APM component expression in many types of solid tumors showed that these defects are present in all types of solid tumors tested with a frequency of at least 40%; in some cancer types the frequency of HLA class I APM defects can be as high as 75% [122]. These defects have functional relevance, since they cause resistance of cancer cells to cognate T cells’ recognition and lysis [123] and have clinical relevance in at least some cancer types [122]. In most cases HLA class I APM component downregulation or loss is associated with poor clinical course of the disease. This association has been suggested to reflect the escape of malignant cells from immune surveillance. Defective HLA class I-tumor antigen derived peptide processing, HLA synthesis and complex assembly can cause abnormalities in expression of HLA class I APM components and/or function. As a result, the malignant cell recognition by cognate T cells is defective. Furthermore, in a limited number of patients (mostly melanoma) who respond to T cell-based immunotherapy, disease recurrence was associated with HLA class I APM component down regulation/loss [124]. However, in a few cancer types high expression of HLA class I has been found to be associated with poor prognosis [122]. This unexpected association has been suggested to reflect more important role of NK cells than that of T cells in the control of tumor growth in these malignancies. High HLA class I expression by tumor cells can inhibit the anti-tumor activity of NK cells due to interaction of killer inhibitory receptors (KIR) on NK cells with HLA class I that is a KIR-ligands.

Different types of HLA class I defects have been identified in malignant cells:

1. Total HLA class I antigen loss to selective loss of the HLA class I allospecificities encoded in the genome of a tumor bearing patient. The latter include loss of the gene products of one or two HLA class I loci or of the HLA class I allospecificities encoded by the genes present in the major histocompatibility complex region of one of the parental chromosomes 6.
2. Down-regulation represents the most frequent type of abnormality in APM components, as complete lack of expression has been described only for a few APM components such as TAP1 and tapasin, and for each of them in a few cases. Since expression of the HLA class complex components is determined by the co-dominance of the two HLA genes (one of paternal and the other one of maternal origin) as well as β2m, multiple factors may contribute to the level of the HLA I complex expression.
3. Structural mutations of the genes encoding HLA class I APM components have been found at most in 5% of the cases. Structural abnormalities in β2m which caused lack of HLA class I antigen expression by tumor cells have been reported to be associated with resistance to immune checkpoint inhibitors. A patient with advanced melanoma who acquired resistance to the anti-PD-1 antibody, pembrolizumab, harbored a homozygous β2m truncating mutation [125]. In addition, β2m alterations have also been described in two brain metastases from patients with mismatch repair deficient colorectal cancer who were resistant to immune check point-based therapy [126].
4. Epigenetic mechanisms appear to be the most frequent cause of defects in HLA class I APM component expression [122]. They include histone acetylation defects and methylation of the promoters of the genes encoding HLA class I APM components. Strategies to counteract the epigenetic mechanisms have been developed. They include the use of histone acetylation inhibitors and demethylating agents. These approaches have been shown to restore HLA class I APM component expression and/or function in vitro, in animal model systems and/or in clinical settings. These results emphasize the need to characterize the expression and function of HLA class I APM in tumors from patients who are resistant or acquire resistance to immune checkpoint inhibitor therapy. The resulting information may contribute to the successful design of therapies which combine immune checkpoint inhibitors with strategies which restore the expression and/or function of HLA class I APM components. This combinatorial therapy is expected to restore patients’ sensitivity to immune checkpoint inhibitor therapy at least in some cases and to enhance the efficacy of this type of therapy in others.

Despite the potential role of HLA class I APM in the response to therapy with checkpoint inhibitors, only a limited number of studies has correlated the expression of HLA class I APM components in tumors with response to immune checkpoint inhibitor therapy or has analyzed the expression of HLA class I APM components in tumors from patients who have not responded to this type of therapy or have acquired resistance to it. However, studies begin to analyze the role of interactions between HLA class I antigens and checkpoint molecules in the clinical course of the disease. High HLA class I antigen expression in intrahepatic cholangiocarcinoma and in esophageal cancer has been found to be associated with good prognosis only when PD-L1 is not detected on tumor cells [127, 128]. One might argue that when PD-L1 binds to PD-1 on cognate T cells and inhibits their anti-tumor activity, HLA class I antigen expression may not play a major role in the interactions of tumor cells with cognate T cells. In contrast, when PD-L1 is not expressed on tumor cells and cognate T cells are able to attack tumor cells, HLA class I antigen expression is necessary to recognize and kill malignant cells. The latter scenario is likely to mimic what happens in the clinical setting when the PD-1/PD-L1 axis is disrupted with inhibitors. These findings imply that selection of patients to be treated with immune checkpoint inhibitors should consider the expression of HLA class I APM components by the tumor cells. When this machinery is not fully functional, HLA class I independent immunotherapeutic strategies appear to be the strategies of choice.

A word of caution about the absolute requirement of a fully functional HLA class I APM in tumor cells for response to anti-PD-1 therapy. About 70% of patients with Hodgkin disease carry β2m-inactivating mutations in their tumor cells, which cause lack of HLA class I expression [129]. Nevertheless, these patients show dramatic response rates to anti-PD-1 therapy [130]. Whether this surprising result reflects the targeting of HLA class II antigen bearing tumor cells by CD4 cells remains to be determined.

### Prognostic and predictive markers for patients treated with CTLA-4 and PD-1 inhibitors

Recently it was demonstrated that patients who discontinue nivolumab in combination with ipilimumab due to drug toxicity derive an OS benefit similar to that observed in the overall population at 18-month follow-up [131]. Different diagnostic tests have been approved to determine PD-L1 expression in tumor, with different cut-off positivity. Response by PD-L1 expression level (5%) in Checkmate067 showed that patients with PD-L1 positive tumors treated with nivolumab in combination with ipilimumab had higher ORR [132].

A recent study evaluated the expression of PD-L1 in immunotherapy-naïve metastatic melanoma patients and demonstrated that PD-L1 expression was frequently discordant between primary tumors and metastases as well as between intrapatient metastases [133]. A positive univariate association between PD-L1 expression in locoregional metastases and melanoma-specific survival was observed, but not for primary melanoma. In locoregional lymph node metastasis, PD-L1+/TIL+ patients had the best outcome, and PD-L1+/TIL− patients had poor outcome [133].

Studies of survival rate and PD-L1 expression in the CheckMate 066 trial demonstrated that, patients treated with nivolumab had improved overall survival regardless of PD-L1 status, as compared with dacarbazine-treated patients however the median overall survival was not reached in either PD-L1 subgroup treated with nivolumab. In the dacarbazine group, the median overall survival was slightly longer in the subgroup with positive PD-L1 as compared with the subgroup with negative or indeterminate PD-L1 status.

The assessment of biomarkers associated with clinical outcome following ipilimumab treatment in advanced melanoma patients showed that low Serum Lactate Dehydrogenase (LDH), absolute monocyte counts (AMC), and MDSCs as well as high absolute eosinophil counts (AEC), Tregs, and relative lymphocyte counts (RLC) is associated with favorable outcome following ipilimumab [134]. Patients (43.5%) presenting with the best biomarker signature (a baseline signature of low LDH, AMC, and MDSCs as well as high AEC, Tregs, and RLC) had a 30% response rate and median survival of 16 months. In contrast, patients with the worst biomarkers (27.5%) had only a 3% response rate and median survival of 4 months [135].

The impact of γδ T-cells on OS and of the first dose of ipilimumab in melanoma patients was assessed in 109 melanoma patients 109 healthy controls [136]. Patients with higher frequencies of Vδ1+ cells (≥ 30%) had poorer OS (p = 0.043). In contrast, higher frequencies of Vδ2+ cells (≥ 39%) were associated with longer survival (p = 0.031) independent of the M category or lactate dehydrogenase level. Besides, frequencies of both Vδ1+ and Vδ2+ cells demonstrated to be candidate biomarkers for outcome in melanoma patients following ipilimumab [136].

Changes in blood counts and frequency of circulating immune cell populations analyzed by flow cytometry were investigated in 82 patients to compare baseline values with different time-points after starting ipilimumab. Endpoints were OS and best clinical responses [134]. ALC increases at 2–8 weeks (p = 0.003) and CD4+ and CD8+ T cells 8–14 weeks (p = 0.001 and p = 0.02) after the first dose of ipilimumab were correlated with improved survival and with clinical responses (all p < 0.05). Early increase of ALC and delayed increase of CD4+ T cells and early increase of ALC and delayed increase of CD4+ and CD8+ T cells were also significant [134].

LDH, routine blood count parameters, and clinical endpoints OS and best overall response following pembrolizumab treatment were investigated in 616 patients [137]. The biomarkers studied for pembrolizumab, by multivariate model with Cox regression analysis for overall survival, were the pattern of visceral metastases; serum levels of LDH; relative lymphocyte count; and relative eosinophil count. Biomarkers showed independent positive correlation (all p < 0.001), that was subsequently confirmed in the validation cohort (n = 257; all p < 0.01). The probability to survive 12 months after the first dose was 15% in patients with none out of four favorable factors, in contrast to 84% for patients with all four favorable factors present. Because of the large differences in outcome according to the number of favorable factors, it remains unclear whether the combination model is predictive of pembrolizumab treatment benefit [137].

Table 3 summarizes the prognostic and predictive markers for checkpoint inhibitors.

**Table 3:** Biomarkers for checkpoint inhibitors: prognostic (in italics) and predictive details

Ipilimumab	Pembrolizumab
*Lactate dehydrogenase*	*Stage III-IVB/IVC*
Relative lymphocyte counts (RLC)	*Lactate dehydrogenase (LDH)*
Absolute eosinophil counts (AEC)	Relative lymphocyte counts (RLC)
Absolute monocyte counts (AMC)	Relative eosinophil counts (REC)
Tregs: unknown	
MDSC: unknown	
γδ T-cells: uncertain	
Increase CD4+ CD8+ T cell: uncertain	

The presence of circulating T cells responding to Melan-A or NY-ESO-1 had strong independent prognostic impact on survival in advanced melanoma, suggesting that these could be targets for immunotherapy [138]. In cohort A patients (84 patients with follow-up after analysis), the presence of T cells responding to peptides from NY-ESO-1, Melan-A, or MAGE-3 and the M category were significantly associated with survival. NY-ESO-1 and Melan-A (hazard ratios, 0.29 and 0.18, respectively) remained independent prognostic factors in Cox regression analysis and were superior to the M category in predicting outcome [138]. Median survival of patients possessing T cells responding to NY-ESO-1, Melan-A, or both was 21 months, compared with 6 months for all others. Melan-A responses were found in 42 and 47% of patients in cohort A and B (24 months survival after first occurrence of distant metastases; cohort B), respectively. In contrast, the proportion was only 22% for NY-ESO-1 and 23% for Melan-A in those who died within 6 months [138].

Identifying peptides recognized by individual T cells is important for understanding and treating immune-related responses. A peptide–Major Histocompatibility Complex (MHC) multimers labeled with individual DNA barcodes to screen > 1000 peptide specificities in a single sample that detect low-frequency CD8 T cells specific for virus- or cancer-restricted antigens was investigated [139]. Several neoepitope-specific T cells in tumor-infiltrating lymphocytes were identified. Barcode-labeled pMHC multimers enable the combination of functional T-cell analysis with large-scale epitope recognition profiling to characterize T-cell recognition in various diseases, including in clinical patients samples [139].

In conclusion, prognostic and predictive markers for patients treated with CTLA-4 and PD-1 inhibitors are:

1. Clinical outcome of first shot which may be the most relevant biomarker.
2. Established prognostic markers like tumor-stage and LDH which are valid in checkpoint inhibitor therapy.
3. Lymphocyte and eosinophil counts which may have predictive value.
4. Preexisting functional antitumor T cell responses which should be better analyzed.

### Update on PD-L1 and related markers

FDA approvals for immune checkpoint blockade agents have been numerous both in melanoma and other tumors. A number of these approvals have been accompanied by immunohistochemical (IHC) assays for PD-L1 in the form of complementary or companion diagnostics (CDxs).

Complementary diagnostic for melanoma was approved approximately 5 years after anti-CTLA-4 was first approved, and as such, practitioners were well-accustomed to administering checkpoint blocking agents to patients with melanoma without testing for PD-L1 expression. This is in contrast with non-small cell lung carcinoma, where approvals for the agent itself were often accompanied by approval for the Companion or Complementary diagnostic.

The publicly available data on PD-L1 IHC testing in patients with metastatic melanoma as of October 2015, shows that practitioners tested approximately 37% of their patients for PD-L1 expression vs. testing 98% of patients for BRAF mutations (Source: BrandImpact/BrandImpact Dx). When melanoma patients were tested, only 20% of surgical pathology labs were running CDxs, with more labs either running a laboratory developed tests (LDT) for PD-L1 IHC or sending the test out to be performed by a Reference Laboratory. Since the PD-L1 IHC Complementary Dx for melanoma was approved by FDA after this survey was conducted, it is possible that the proportion of oncologists ordering PD-L1 testing as well as surgical pathology labs performing the test will increase in subsequent surveys.

There are numerous commercially available PD-L1 immunohistochemistry (IHC) assays (Table 4). It is impractical for most surgical pathology laboratories to host four different IHC assays for PD-L1, thus there is great interest in understanding the comparative performance of these assays.

**Table 4:** Commercially available PD-L1 IHC assays

	BMS	Merck	Roche	Astra Zeneca
mAb clone	28-8	22C3	SP142	SP263
Automated	Yes	Yes	Yes	Yes
Diagnostic partner	Dako	Dako	Ventana	Ventana
Machine	Link 48	Link 48	Benchmark ULTRA	Benchmark ULTRA
Scoring	Tumor cells (membrane)	Tumor cells (membrane)	Tumor cells/or immune (membrane)	Tumor cells (membrane)
Positive cutoff	≥ 5% (also studied ≥ 1% and ≥ 10% thresholds	≥ 1% for trial enrollment Other analysis at > 50%	TC3 (> 50%) or IC3 (> 10%) TC2/3 or IC2/3 (> 5%) TC 1/2/3 or IC 1/2/3 (> 1%) TC0 and IC0 (0%)	≥ 25%

The scoring systems noted below often depend on the line of therapy and the tumor type being tested. An increasing number of assays from different companies are reaching the market

To that aim, the Blueprint PD-L1 IHC Assay Comparison Project was initiated. It is an industrial-academic collaborative partnership to provide information on the analytical performance of these four PD-L1 IHC assays [140]. Phase 1 was conducted by staining n = 40 NSCLC with each of the four PD-L1 IHC assays (using clones 22C3, 28-8, SP142, and SP263). Both the percent of tumor cells and immune cells staining for PD-L1 were assessed by three pathologists, and their scores were averaged. Three of the four assays (22C3, 28-8, and SP263) were closely aligned on tumor cell staining whereas the fourth (SP142) showed consistently fewer tumor cells stained. All the assays demonstrated immune cell staining, but with greater variability than with tumor cell staining.

A second study tested 90 archival NSCLCs stained by different PD-L1 IHC assays and scored by 13 pathologists. The assays tested in this second study included: (1) 28-8 assay on Dako Link 48; (2) 22c3 assay on Dako Link 48; (3) SP142 assay on Ventana Benchmark (LDT mimicking the IUO test); and (4) E1L3N antibody on Leica Bond (LDT). Similar to the Blueprint study, the assay using the SP142 antibody was an outlier detecting significantly less tumor cell PD-L1 expression. It was also an outlier with respect to detecting immune cell PD-L1 expression [141].

To determine whether the performance characteristics of the SP142 assays were due to the SP142 antibody itself or to the assay conditions, we conducted a study where we held all of the other assay conditions (antigen retrieval, buffers, amplification systems) essentially constant and changed only the antibody. In this study, we assessed archival melanoma cases, and compared the 5H1, SP142, SP263, 22C3, and 28-8 antibody clones. We found that all antibodies could demonstrate similar staining properties regarding labeling PD-L1 display by both tumor cells and immune cells, if all other assay conditions were constant. These findings indicate that it is likely the assay conditions (beyond the primary antibody itself) that contribute to the differences observed between the SP142 assay and the other studied assays [142].

In addition to PD-L1, there is a number of other candidate biomarkers which have been nominated, including PD-1, CD8, and mutational load. PD-L2 has not been well-studied yet, due to the lack of well-validated commercially available antibodies. We next used the TCGA dataset to assess how these different markers related to PD-L1 expression. We found that PD-L1, PD-L2, PD-1, cytolytic activity (CYT), and mutational load are all positive prognostic features in patients with melanoma. Notably, PD-L1, PD-L2, PD-1 and CYT are all closely linked to each other and to Th1/IFNγ expression in patients with melanoma, while mutational load is not. When principal component analysis was conducted on these five variables, PD-L1, PD-L2, PD-1 and CYT all formed the first principal component, with each contributing near equally, while mutational load was orthogonal and formed the second principle component. We then assessed the relative contribution of these factors to survival using conditional inference modeling, which revealed that PD-1/CYT expression (i.e., an inflamed tumor microenvironment) as the most impactful feature, followed by mutational density in tumors that were less inflamed [143].

Additional studies are underway to perform similar analyses as they relate to predicting response or resistance to checkpoint blockade, as well as further refining prognostic algorithms. While genomic studies and gene signatures contribute global assessments of the tumor microenvironment, emerging data reinforces the contribution of spatially-resolved approaches to protein expression. Future biomarker panels for patients with melanoma will likely include multiplex IHC/immunofluorescent studies as well as genomic studies and gene signatures. Assay development will thus require the integration and prioritization of these different modalities.

### Understanding responses to cancer therapy: lessons learned from mouse and man

The Cancer Genome Atlas (TCGA) program performed a systematic multi-platform characterization of 333 cutaneous melanomas at the DNA, RNA, and protein levels to create a catalog of somatic alterations and describe their potential biological and clinical significance. TCGA represents the largest integrative analysis of cutaneous melanoma; it establishes a framework for melanoma genomic classification (BRAF, RAS, NF1, and Triple-WT), it identifies additional subtypes that may benefit from MAPK-and RTK-targeted therapies and multi-dimensional analyses identify immune signatures associated with improved survival [144].

Therapeutic targeting of oncogenic BRAF mutations results in rapid regression of melanoma tumors in most patients. Clinical trials showed that treatment with BRAF inhibitors resulted in a survival benefit over then standard of care therapy with dacarbazine substantiating the FDA approval of the first of these agents (vemurafenib) in 2011. However, most of patients progressing on therapy within 6 months that is the limitations of this therapy. Insight into resistance mechanisms has led to advances in therapy, including the use of combined BRAF and MEK inhibition, which was shown to improve progression free survival in patients; even with the combination, most of patients progress within a year. There is a critical need to identify pre-treatment biomarkers of response and resistance, as well as early on-treatment markers of resistance which are potentially actionable.

Substantial research efforts internationally focused on response and resistance to targeted therapy for melanoma in biopsies of patients treated with BRAF inhibitors. The team of researchers at the MD Anderson CC performed genomic analysis in pre-treatment and progressing lesions, with immune profiling in pre-treatment, on-treatment and progressing lesions. Through such studies, multiple genomic mechanisms of resistance have been identified and published.

Although oncogenic mutations contribute to tumor escape via multiple mechanisms, including through immune evasion mutations can make tumors more immunogenic. Therefore, in addition to studying genomic mechanisms of resistance, immune profiling was also performed in tumors over the course of therapy to gain insights into anti-tumor immunity was researched out. Specifically, T cell infiltrate and markers of cytotoxicity, the expression of immunosuppressive cytokines and VEGF as well as PD-1 and PD-L1 were assessed. Through these studies, immune mechanisms of response and resistance to targeted therapy were also identified. Treatment with BRAF-targeted therapy increased melanoma antigens expression (e.g., MART) and CD8+ T cells infiltrate resulting in more favorable TME. Furthermore, decreased the level of immunosuppressive cytokines and VEGF, within 2 weeks of starting therapy providing support for potential synergy of BRAF-targeted therapy and immunotherapy [69]. However along with these favorable changes, an increase in expression of the immunomodulatory molecule PD-L1 early during treatment was noted, suggesting a possible immune mechanism of resistance to therapy. Interestingly, these favorable immune effects are not likely to be solely related to increased melanoma antigens but to overall changes in the tumor microenvironment. Hypothesis that combining targeted therapy and immune checkpoint blockade would enhance responses to therapy was tested in mouse models. A synergy was found in these studies, with delayed tumor outgrowth and prolonged survival when mice were treated with BRAF targeted therapy and PD-1 blockade, compared to either therapy alone [70]. There was a modest CD8 T cell infiltrate in the setting of BRAF inhibitor monotherapy, but when PD-1 blockade was added to a backbone of a targeted therapy a dramatic increase in CD8 T cell infiltrate in the tumors was observed [70]. Based on this observation, multiple trials are underway combining these strategies for melanoma as well as other cancer.

In addition, these treatments are also being assessed in patients with earlier stage disease. Upfront surgery is the standard treatment for patients with clinical stage III melanoma. However, most patients treated with upfront surgery will relapse and die (~ 70%). A phase 2 clinical trial to test the hypothesis that treatment with neoadjuvant and adjuvant BRAF/MEK inhibitors would improve RFS in patients with bulky stage III melanoma and a BRAF mutation (vs upfront surgery and standard of care adjuvant therapy) is currently underway, and translational assessments to identify molecular and immune determinants of response are ongoing [145].

Furthermore, mechanisms of responses to immune checkpoint blockade targeting CTLA-4 and PD-1 through translational research in human samples are also studied. Not all patients respond to immune checkpoint inhibitor therapy particularly as monotherapy. Tremendous efforts are underway to identify predictive biomarkers of response to immune checkpoint blockade based in tumor biopsies including mutational load at baseline with evidence that patients with a higher mutational load have improved responses to checkpoint inhibitor therapy [47] though this is not perfectly predictive and better biomarkers are needed. In addition, immune markers are being studied including CD8 T cell density and distribution as well as IFNγ response gene signatures [96, 97].

Cohort of patients with metastatic melanoma who initially received Ipilimumab and then went onto PD-1 blockade therapy, and isolated tissue samples from multiple time points on therapy and embarked on a deep molecular and immune profiling of these tumors was carried out [146]. In this study, immune signatures in pre-treatment tumor biopsies assessed by a 12-marker immunohistochemistry panel largely failed to predict response to PD-1 blockade, but signatures in on-treatment biopsies were highly predictive [146]. Gene expression profiling using Custom 795 gene NanoString set was used to see if responses to immune checkpoint blockade can be predicted via gene expression profiling. Gene expression profiles at pre-treatment time point largely failed to predict response to immune checkpoint blockade. However, signatures in on-treatment PD-1 samples were strongly predictive [146].

These data suggest that clinically useful biomarkers for response to immune checkpoint blockade exist, but specific time point to assess them might be critical. The study needs to be validated in additional cohorts, the emphasis, should rather be on assessing adaptive immune responses in early on-treatment samples for response to checkpoint therapy (and should be incorporating this into clinical trials) instead on pre-treatment biomarkers. Importantly, we are also bringing immune checkpoint inhibitor treatment to patients with earlier stage disease, and have an ongoing phase 2 trial to test the hypothesis that treatment with neoadjuvant (+ adjuvant) immune checkpoint inhibitors will be associated with a high pathological complete response (path CR) rate and immune infiltrate (NCT02519322). In this trial, patients with stage IIIB or stage IIIC melanoma are randomized to treatment with PD-1 monotherapy versus combined CTLA-4 and PD-1 blockade. Preliminary findings are available [147] though mature data will not be available for some time.

In addition to the influence of the tumor and microenvironment in modulating responses to therapy in melanoma and other cancers, there is a growing role of environmental factors that may contribute to therapeutic response and resistance. Pioneering work by Gajewski and Zitvogel demonstrated that bacteria in the gut could modulate therapeutic responses to immune checkpoint blockade in pre-clinical models [107, 148]. We recently studied this in a cohort of patients with metastatic melanoma, and found that differential bacterial signatures existed in responders versus non-responders to immune checkpoint blockade (specifically anti-PD-1 therapy), and reported these findings at the annual meeting of the American Society of Clinical Oncology in 2017. Additional studies are currently underway to better understand the mechanism behind this, and to develop strategies to enhance therapeutic responses via modulation of the microbiome.

Another innovative technique is the use of Raman spectroscopy, a noninvasive and label-free optical technique that provides detailed information about the molecular composition of a sample. It can potentially predict skin toxicity due to tyrosine kinase inhibitors treatment. Raman spectra of skin of patients undergoing treatment with MEK, EGFR, or BRAF inhibitors, which are known to induce severe skin toxicity were collected (three patients were included for each inhibitor) [149]. The algorithm, based on partial least squares-discriminant analysis (PLS-DA) and cross-validation by bootstrapping, can identify patients with risk for cutaneous adverse events. For MEK and EGFR inhibitors, discriminative power was more than 90% in the viable epidermis skin layer; whereas for BRAF inhibitors, discriminative power was 71% [149]. Raman spectroscopy can detect skin toxicity induced by TKI treatment on locations not affected from a dermatological and histological point-of-view. It has better discriminative power in the case of MEK and EGFR inhibitors which are known to be associated with inhibition of MAPK pathway on healthy tissue contrary to BRAF inhibitors. There is good correlation (> 80%) of Raman signature of the skin and drug concentration into the blood in the case of EGFR inhibitors and VE skin layer.

## Abbreviations

ALC: absolute lymphocyte counts
ATP: adenosine triphosphate
AFP: alpha-fetoprotein
BRAFi: BRAF inhibitors
COG’s: Children’s Oncology Group
CR: complete response
CYT: cytolytic activity
DCs: dendritic cells
IDO: indolamine 2,3 dehydrogenase
DRR: durable response rate
GCT: germ cell tumor
HLA: human leukocyte antigen
HIF: hypoxia-inducible factor
ICB: immune checkpoint blockers
ICD: immunogenic cell death
IHC: immunohistochemistry
IFN-I: interferon type I
IEC: intestinal epithelial cells
LDH: lactate dehydrogenase
MHC: major histocompatibility complex
miRNAs: microRNAs
mAb: monoclonal antibody
NK: natural killer cells
NGS: next generation sequencing
ORR: objective response rate
PBMCs: peripheral blood mononuclear cells
RT: radiotherapy
T-VEC: talimogene laherparepvec
TIL: tumor infiltrating lymphocytes
TME: tumor micro environment
WGCNA: weighted gene coexpression network analysis
